# RC3H1 post-transcriptionally regulates A20 mRNA and modulates the activity of the IKK/NF-κB pathway

**DOI:** 10.1038/ncomms8367

**Published:** 2015-07-14

**Authors:** Yasuhiro Murakawa, Michael Hinz, Janina Mothes, Anja Schuetz, Michael Uhl, Emanuel Wyler, Tomoharu Yasuda, Guido Mastrobuoni, Caroline C. Friedel, Lars Dölken, Stefan Kempa, Marc Schmidt-Supprian, Nils Blüthgen, Rolf Backofen, Udo Heinemann, Jana Wolf, Claus Scheidereit, Markus Landthaler

**Affiliations:** 1 RNA Biology and Posttranscriptional Regulation, Berlin Institute of Medical Systems Biology at the Max-Delbrück Center for Molecular Medicine, 13125 Berlin, Germany; 2 Signal Transduction in Tumor Cells, Max-Delbrück Center for Molecular Medicine, 13125 Berlin, Germany; 3 Mathematical Modelling of Cellular Processes, Max-Delbrück Center for Molecular Medicine, 13125 Berlin, Germany; 4 Macromolecular Structure and Interaction, Max-Delbrück Center for Molecular Medicine, 13125 Berlin, Germany; 5 Helmholtz Protein Sample Production Facility, Max Delbrück Center for Molecular Medicine, 13125 Berlin, Germany; 6 Department of Computer Science and Centre for Biological Signalling Studies (BIOSS), Albert-Ludwigs-Universität Freiburg, 79110 Freiburg, Germany; 7 Immune Regulation and Cancer, Max-Delbrück Center for Molecular Medicine, 13125 Berlin, Germany; 8 Integrative Proteomics and Metabolomics Platform, Berlin Institute of Medical Systems Biology at the Max-Delbrück Center for Molecular, 13125 Berlin, Germany; 9 Institut für Informatik, Ludwig-Maximilians-Universität, 80333 München, Germany; 10 Institute for Virology and Immunobiology, University of Würzburg, 97078 Würzburg, Germany; 11 Department of Hematology and Oncology, Technische Universität, 81675 München, Germany; 12 Institute of Pathology, Charité–Universitätsmedizin Berlin, 10117 Berlin, Germany; 13 Integrative Research Institute (IRI) for the Life Sciences and Institute for Theoretical Biology, Humboldt-Universität zu Berlin, 10115 Berlin, Germany; 14 Chemistry and Biochemistry Institute, Freie Universität Berlin, 14195 Berlin, Germany

## Abstract

The RNA-binding protein RC3H1 (also known as ROQUIN) promotes TNFα mRNA decay via a 3′UTR constitutive decay element (CDE). Here we applied PAR-CLIP to human RC3H1 to identify ∼3,800 mRNA targets with >16,000 binding sites. A large number of sites are distinct from the consensus CDE and revealed a structure-sequence motif with U-rich sequences embedded in hairpins. RC3H1 binds preferentially short-lived and DNA damage-induced mRNAs, indicating a role of this RNA-binding protein in the post-transcriptional regulation of the DNA damage response. Intriguingly, RC3H1 affects expression of the NF-κB pathway regulators such as IκBα and A20. RC3H1 uses ROQ and Zn-finger domains to contact a binding site in the A20 3′UTR, demonstrating a not yet recognized mode of RC3H1 binding. Knockdown of RC3H1 resulted in increased A20 protein expression, thereby interfering with IκB kinase and NF-κB activities, demonstrating that RC3H1 can modulate the activity of the IKK/NF-κB pathway.

## Abstract (web summary)

The RNA-binding protein RC3H1/ROQUIN1 promotes the degradation of mRNA by binding to a consensus CDE present in the 3′UTR. Here the authors expand the set of consensus sequences through which RCH31 binds and regulates mRNA encoding members of the DNA damage response and IKK/NF-κB pathway.

## Graphical abstract

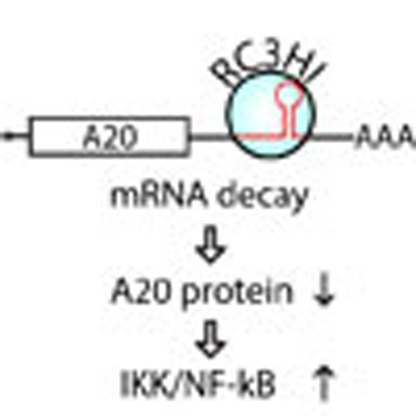

Post-transcriptional regulation of gene expression by RNA-binding proteins (RBPs) controls a variety of cellular processes. Especially, the modulation of messenger RNA (mRNA) stability is of critical importance for the dynamic regulation of genes such as transcription factors and cytokines that need to be switched on and off rapidly^1^,^2^.

Roquin is an RBP with a central role in repressing autoimmunity^3^. Originally, a missense mutation in the *Rc3h1* gene encoding the Roquin-1 protein was identified as the cause of systemic lupus erythematosus-like autoimmunity phenotype in *sanroque* mice^3^. Roquin-1 is localized in cytoplasmic granules^4^,^5^ and binds to the 3′ untranslated region (3′UTR) of inducible costimulator (ICOS) mRNA to post-transcriptionally repress its expression^6^,^7^. Furthermore, Roquin-1, as well as its paralogue Roquin-2, interacts with 3′UTR of TNFRSF4 and tumour-necrosis factor-α (TNFα), and modulates immune responses^5^,^8^. Recent studies showed that Roquin proteins interact through their ROQ domains with a constitutive decay element (CDE) in the 3′UTR of TNFα mRNA and promotes the decay of this transcript by recruiting the CCR4-CAF1-NOT deadenylase complex^9^. The CDE of TNFα folds into a characteristic stem–loop structure containing a specific trinucleotide loop, which is highly similar to the Roquin-1 RNA recognition element in the ICOS 3′UTR (ref. 9). Latest structural analyses showed the ROQ domain in complex with a prototypical CDE RNA stem–loop revealing recognition of the RNA stem and its trinucleotide loop^10^,^11^. Leppek *et al.*^9^ further identified additional Rc3h1 target transcripts by RNA-immunoprecipitation sequencing (RIP-seq) analysis, including regulators of the nuclear factor-κB (NF-κB) pathway. However, a recognizable CDE was absent in the majority of Rc3h1-bound mRNAs, suggesting other modes of RNA recognition^9^. In line with these findings, Schlundt *et al.*^10^ showed by mutational and structural analyses of RNA ligands that relaxed CDE consensus sequences can mediate Roquin-dependent regulation. Similarly, Tan *et al.*^11^ and Schuetz *et al.*^12^ reported that the ROQ domain of Rc3h1 recognizes the CDE and can also bind to duplex RNA. In addition to the ROQ domain, RC3H1 possesses an N-terminal RING finger with a potential E3 ubiquitin–ligase function^13^, as well as a CCCH-type zinc (Zn) finger that is involved in RNA recognition^7^. CCCH-type Zn-finger RBPs typically contact AU-rich elements^14^,^15^. AU-rich elements are conserved *cis*-regulatory elements, originally discovered in the 3′UTRs of short-lived mRNAs, encoding inflammatory mediators^16^,^17^,^18^.

To obtain a better understanding of the molecular mechanisms of human RC3H1 RNA recognition and disentangle its cellular function, we applied PAR-CLIP (photoactivatable ribonucleoside-enhanced crosslinking and immunoprecipitation)^19^ to identify RC3H1 RNA-binding sites and target transcripts in HEK293 cells. RC3H1 contacts mRNAs through structure-sequence elements located in 3′UTRs. The binding sites are composed of hairpins with variable loop length often with embedded U-rich sequences, including CDE consensus sequences. RC3H1-bound mRNA targets are short-lived, and RC3H1 depletion results in decreased mRNA decay rates and increased protein synthesis of its target mRNAs. RC3H1 target transcripts are enriched for mRNAs that are induced upon DNA damage, among them are the mRNA of A20 (also known as TNFAIP3). A20 codes for an ubiquitin-editing enzyme, which inhibits activation of NF-κB^20^,^21^. *In vitro* and *in vivo* experiments revealed that RC3H1 interacts with a non-CDE-type stem–loop structure preceded by an AU-rich sequence in the A20 3′UTR involving ROQ and CCCH-type Zn-finger domains, indicating a yet unrecognized RC3H1-binding mode and specificity. Depletion of RC3H1 leads to increased A20 protein expression, which is accompanied by decreased IκB kinase (IKK) activation and NF-κB DNA-binding activity upon TNFα signalling.

## Results

### Human RC3H1 binds to thousands of mRNAs

**Figure 1:**
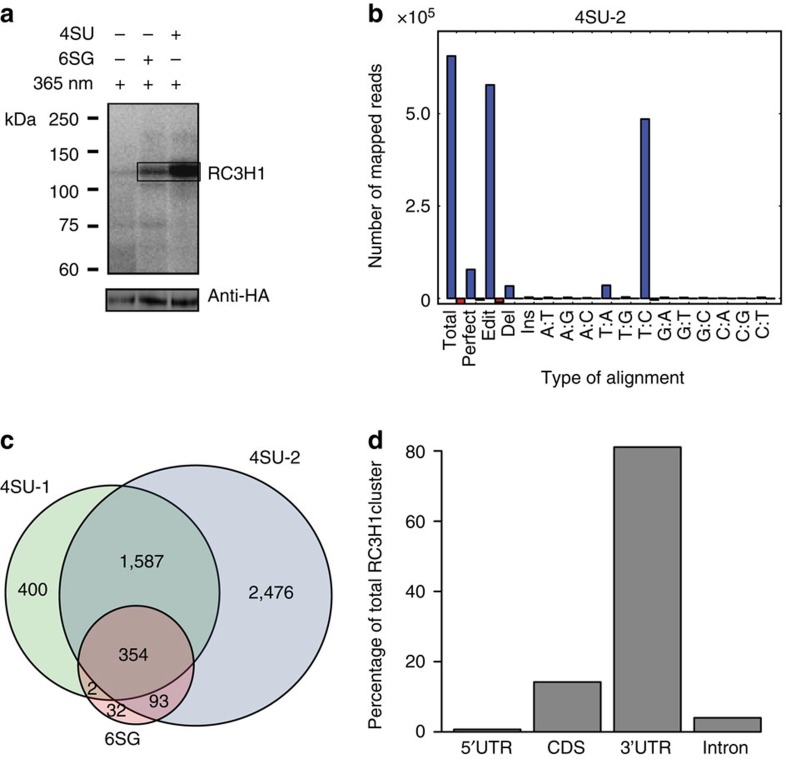
PAR-CLIP identifies thousands of human mRNAs directly bound by RC3H1. (**a**) Phosphorimage of SDS–PAGE of radiolabelled FLAG/HA-RC3H1–RNA complexes from 365 nm ultraviolet light crosslinked non-labelled, 6SG- or 4SU-labelled cells. Crosslinked protein–RNA complexes were observed upon metabolic labelling with 4SU or 6SG. The lower panel shows an anti-HA western blot, confirming correct size and equal loading of the IPed protein. The box indicates the region that was cut out for PAR-CLIP library preparation. (**b**) Specific T to C mismatches in aligned reads demonstrate efficient mRNA-RBP crosslinking. The frequency of nucleotide mismatches in 4SU-2 PAR-CLIP reads aligned to mature mRNAs is shown. Sense mapping is shown in blue and antisense mapping in red. (**c**) A Venn diagram showing the overlap of target mRNA transcripts between 4SU and 6SG PAR-CLIP experiments. (**d**) Distribution of binding sites along mRNA transcripts based on consensus RC3H1 PAR-CLIP-binding sites. The majority of binding sites are located in 3′UTRs. CDS, coding sequences.

To identify RC3H1-binding sites at high resolution, we applied PAR-CLIP in combination with next-generation sequencing^19^. In PAR-CLIP experiments, nascent RNA is metabolically labelled with the non-perturbing photoreactive ribonucleosides 4-thiouridine (4SU) or 6-thioguanosine (6SG). Crosslinking of protein to 4SU- or 6SG-labelled RNA leads to specific T to C or G to A transitions, respectively, that occur at high frequency in complementary DNA (cDNA) sequence reads and mark the protein crosslinking sites on the target RNA^19^. HEK293 cells stably expressing inducible FLAG/HA-tagged RC3H1 (Supplementary Fig. 1a) were crosslinked after labelling of RNA with either 4SU or 6SG. Immunopurified, ribonuclease-treated and radiolabelled RC3H1–RNA complexes were separated by SDS–polyacrylamide gel electrophoresis (PAGE) (Fig. 1a). Protein-protected RNA fragments were recovered and converted into a cDNA library amenable to Illumina sequencing.

In total, we performed three independent PAR-CLIP experiments (two biological replicates with 4SU, 4SU-1 and 4SU-2, and one replicate with 6SG; see Supplementary Table 1). Sequence reads were mapped to the human genome and overlapping reads were used to build RC3H1-binding clusters^22^. In PAR-CLIP experiments using 4SU, diagnostic T to C transitions detected in mapped reads were most highly abundant (Fig. 1b and Supplementary Fig. 1b). Similarly, but less pronounced, the diagnostic G to A changes were the most abundant type of mutation for the 6SG PAR-CLIP experiment (Supplementary Fig. 1c). A length histogram of RC3H1 PAR-CLIP clusters shows a median cluster size of ∼25–30 nucleotides (Supplementary Fig. 1d).

We identified ∼2,000–4,000 RC3H1 mRNA target transcripts in each of the 4SU PAR-CLIP experiments (Fig. 1c). Ninety-three per cent of the 481 6SG PAR-CLIP mRNA targets were reproduced in 4SU libraries (Fig. 1c). We combined the ‘reads' from all PAR-CLIP experiments to derive a set of consensus binding sites supported by reads from at least two out of three experiments (see Methods section). Based on this analysis, we identified 16,234 RC3H1-binding sites on 3,821 protein-coding transcripts as consensus data set (Supplementary Data 1). The binding sites and PAR-CLIP sequence alignments are publicly available at http://bimsbstatic.mdc-berlin.de/landthaler/RC3H1. The position with the highest number of PAR-CLIP-derived diagnostic nucleotide transitions for each binding sites was referred to as the preferred crosslinking site.

To gain an insight into the transcripts regulated by RC3H1, genes encoding RC3H1-bound mRNAs were subjected to Kyoto Encyclopedia of Genes and Genomes (KEGG) pathway and Gene Ontology (GO) term enrichment analysis^23^,^24^. Interestingly, cell cycle and p53 signalling pathway were overrepresented in the KEGG pathway enrichment analysis (Supplementary Fig. 1e), suggesting that RC3H1 could play a role in the response to DNA damage. Furthermore, GO term enrichment analysis showed that RC3H1-bound transcripts are highly enriched for regulators of gene expression such as transcription factors, RBPs and ubiquitin ligases (Supplementary Fig. 1f). In addition, when comparing the human RC3H1 targets with mouse Roquin-bound transcripts^9^, we identified 36 out of 55 Roquin-interacting mRNAs by PAR-CLIP in HEK293 cells (Supplementary Fig. 1g).

### RC3H1-binding sites are mostly located in 3′UTR of mRNAs

Next, we examined the distribution of RC3H1-binding sites along mRNA transcripts. The majority of binding sites (81%) were found to be located in 3′UTRs (Fig. 1d), consistent with previous observations that RC3H1 binds to ICOS and TNFα mRNAs through 3′UTR interactions^4^,^6^,^7^. RC3H1 binding to ICOS and TNFα mRNAs in HEK293 cells was not observed likely due to undetectable ICOS and TNFα mRNA expression. A preference for RC3H1 binding along 3′UTRs of target transcripts was not apparent, since binding clusters were almost equally distributed over this transcript region (Supplementary Fig. 1h). Since a previous study suggested a functional link between RC3H1 binding and microRNA (miRNA) activity^6^, we examined the local interactions between RC3H1 and miRNA by computing the density of conserved miRNA target sites around RC3H1 preferred crosslinking sites (Supplementary Fig. 1i). The observed profile indicated an overrepresentation of miRNA seed complements in the vicinity of RC3H1-binding sites, but did not directly overlap with these sites.

### U-rich hairpins dominate as recognition features

**Figure 2:**
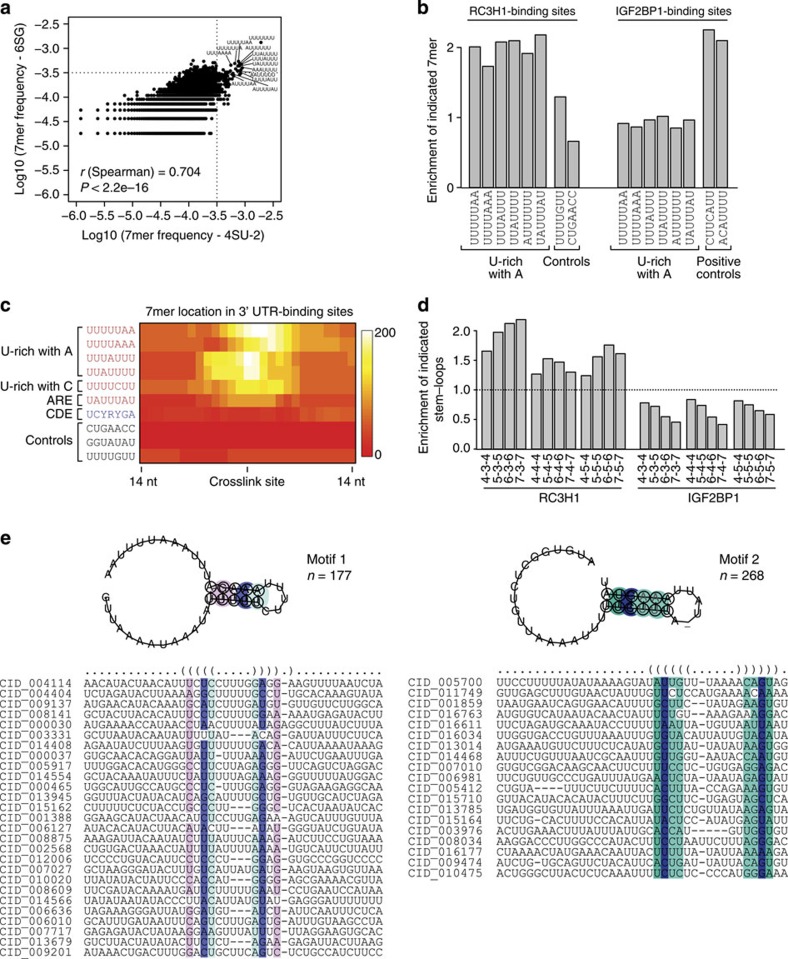
Identification of U-rich sequences and stem–loop secondary structure as recognition elements of RC3H1. (**a**) Log10 frequencies of 7mers occurring in the 41-nucleotide (nt) window around the RC3H1 preferred crosslink sites are shown for 4SU-2 PAR-CLIP and 6SG PAR-CLIP libraries. U-rich sequences are frequently occurring in both 4SU and 6SG libraries. (**b**) Enrichment of indicated 7mers in the 41-nt window around the RC3H1 (left) or IGF2BP1 (right) preferred crosslink sites compared with all 3′UTR sequences. U-rich sequences with A contents are specifically enriched in RC3H1 3′UTR-binding sites, whereas 7mers containing known IGF2BP1 motif (CAU) are enriched in IGF2BP1 3′UTR-binding sites to the similar degree. (**c**) A heat map showing the coverage of 7mers, indicated on the left, around the preferred crosslinks in 3′UTR RC3H1 consensus binding sites. U-rich elements with A contents and CDE are indicated. U-rich sequences are found in the close vicinity of crosslink sites, which is indicative of direct association of RC3H1 with U-rich sequences. (**d**) Enrichment of indicated stem–loop structures in the 41-nt window around the RC3H1 (left) or IGF2BP1 (right) preferred crosslink sites compared with 41 nt sequences randomly selected from the 3′UTRs of target transcripts as a background model. Various stem–loop structures (*n*-*m*-*n* indicates a hairpin structure of *n*-mer stem and *m*-mer loop) are enriched in RC3H1 3′UTR-binding sites but not in 3′UTR IGF2BP1-binding sites. (**e**) The seed alignment and consensus structure of motifs 1 and 2 are shown. ARE, AU-rich elements.

To investigate RNA features recognized by RC3H1, we searched for sequence and secondary structure elements in RC3H1-binding sites. First, we examined 7mer occurrences in 41 nucleotide windows centred on preferred crosslinking sites in RC3H1-binding sites. Notably, U-rich sequences were frequently found in RC3H1-binding sites derived from both 4SU and 6SG experiments (Fig. 2a and Supplementary Fig. 2a), suggesting that the frequent observation of U-richness is not owing to a bias introduced by using 4SU. These U-rich 7mers were overrepresented in RC3H1-binding sites when compared with control 7mers (Fig. 2b). In contrast, U-rich elements were not enriched in IGF2BP1-binding sites^19^, whereas 7mer containing the CAU consensus sequence are overrepresented (Fig. 2b). U-rich sequences with interspersed adenosines were more enriched in RC3H1 consensus binding sites in 3′UTR sequences when compared with U-rich 7mer sequences containing guanosines (Supplementary Fig. 2b and Supplementary Data 2). Similar results were also obtained from 5mer analysis (Supplementary Fig. 2c). In addition, U-rich sequences were found in close proximity of preferred crosslink sites, suggesting the direct interaction of RC3H1 with these sequences (Fig. 2c). In addition, we examined the occurrence of the previously identified CDE motif^9^, and found that the core CDE consensus sequence (UCYRYGA) was present in RC3H1-binding sites, but the frequency was less prominent than that of several U-rich sequences (Fig. 2c).

To examine potential secondary structure features in RC3H1-binding sites, we computationally folded 41-nucleotide sequence stretches centred around the preferred crosslinking sites and averaged the resulting base pairing probabilities. Randomly selected RNA regions of the same length within 3′UTRs of RC3H1 mRNA target transcripts served as a background control. In 3′UTR RC3H1 consensus binding sites, the base pairing probability was reduced in the vicinity of the crosslink sites and increased in the flanking region compared with background, suggesting that RC3H1-binding sites tend to form stem–loop structures (Supplementary Fig. 2d,e). A more detailed examination of the types of hairpin structures enriched in RC3H1 binding revealed an overrepresentation of stems capped by trinucleotide loops as demonstrated for the CDE motif 9,10,11,12, but also hairpins with loops containing 4 and 5 nucleotides (Fig. 2d). From this analysis, we concluded that there might be several binding motifs that are likely to be structured. To test this hypothesis, we applied a variant of an approach that was previously used to detect structured RNA motifs^25^. Here we performed an initial clustering of the set of RNAs, followed by the detection of a specific structure. We started with the top 100 RC3H1 PAR-CLIP-binding sites, and detected initial clusters by LocARNA^26^,^27^ and RNAclust^27^. For each subcluster, we used CMfinder (version 0.2; ref. 28) to search for a subset of sequences that has a specific sequence-structure motif. CMfinder generates both a sequence-structure alignment (referred to as seed alignment) and a covariance model, which we used to search for further sequences in the top 1,000 binding sites for remote members of this motif using cmsearch^29^. The seed alignment of motif 1 (present in 177 out of 1,000 binding sites) and motif 2 (present in 268 out of 1,000 binding sites), representing the most frequently occurring sequence-structure elements, is shown in Fig. 2e. Interestingly, in motif 2, the preferred crosslinked nucleotides are positioned upstream of the predicted hairpin structure, whereas in motif 1 the preferred crosslinked nucleotides are located in the U-rich loop. In summary, our computational analyses did not reveal a defined motif. However, hairpin structures frequently containing U-rich sequences and, albeit, less frequently, the CDE consensus sequence were detected as possible recognition elements of RC3H1.

### RC3H1 interacts with CCR4-CAF1-NOT deadenylase complex

To further investigate the molecular function of RC3H1, we set out to identify proteins that interact with RC3H1. Previous studies showed that RC3H1 destabilizes mRNA albeit by different molecular mechanisms. Glasmacher *et al.*^7^ reported that RC3H1 interacts with mRNA decapping proteins, whereas Leppek *et al.*^9^ more recently showed that roquin protein associates with CCR4-CAF1-NOT deadenylase complex.

**Figure 3:**
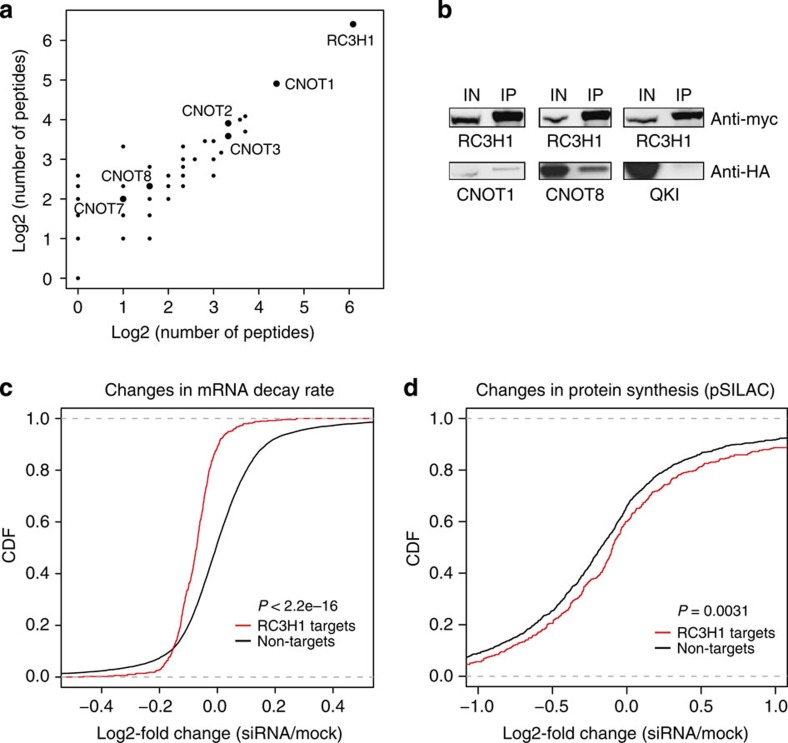
RC3H1 recruits deadenylation complex and destabilizes target mRNAs. (**a**) A scatter plot of identified peptide counts in two label-swap replicates. Peptides eluted from immunopurified FLAG/HA-tagged RC3H1 complex are analysed by tandem LC-MS/MS. High dose of RNaseT1/RNaseI are treated before immunoprecipitation (IP) to disrupt the indirect interactions mediated by nascent RNA. Peptides derived from the CCR4-CAF1-CNOT deadenylase complex were detected. (**b**) RC3H1 interactions were confirmed by co-transfection of myc-RC3H1 expression construct with HA-CNOT1, HA-CNOT8 or HA-QUAKING (QKI) expression constructs. IP was performed using anti-myc antibody. IPed proteins were resolved on SDS–PAGE, blotted and probed with anti-myc and anti-HA antibodies. Protein expression in cellular extract used as input for IP experiments is indicated (IN). (**c**) A cumulative distribution function (CDF) plot of log2-fold changes of mRNA decay rates of the top 500 normalized RC3H1-bound mRNAs is shown in red and all expressed mRNAs is shown in black. Top RC3H1-bound mRNAs show slower mRNA decay rates compared with all mRNAs upon RC3H1/ RC3H2 knockdown. The mean difference in mRNA decay rates (siRNA/mock) for the top 500 RC3H1 target mRNAs (*n*=500) and all mRNAs (*n*=15158) are −1.324 and −0.074, respectively (*P* value <2.2e–16, Wilcoxon's rank sum test). (**d**) A CDF plot of log2-fold changes of protein synthesis of consensus RC3H1 target transcripts that have >100 transitions on 3′UTR is shown in red and non-targets is shown in black after siRNA-mediated RC3H1 depletion. Protein synthesis of RC3H1-bound mRNAs was upregulated upon RC3H1 knockdown (*P* value 0.0031, Wilcoxon's rank sum test). The mean log2-fold changes for RC3H1 targets (*n*=390) and non-targets (*n*=1,279) are 0.001 and −0.116, respectively.

To identify proteins that directly interact with RC3H1 in an RNA-independent manner, cellular extracts of control and FLAG/HA-tagged RC3H1-expressing cells were treated with RNaseT1/I, and the immunoprecipitates were analysed and compared by SILAC (stable isotope labelling by amino acids (aa) in cell culture)-based quantitative mass spectrometry^30^. As a biological replicate, we performed a label-swap experiment with reversed light/heavy isotope labelling (Fig. 3a). As expected, RC3H1 was efficiently immunoprecipitated as indicated by the log2 heavy-to-light normalized ratio of 2.80 in the forward experiment and -5.13 in the reverse experiment (Supplementary Fig. 3a). In two biological replicates, we identified numerous peptides originating from components of the CCR4-CAF1-NOT deadenylase complex including CNOT1, CNOT2, CNOT3, CNOT7 and CNOT8, but not from the decapping complex (Fig. 3a, Supplementary Fig. 3a and Supplementary Table 2). CNOT1 is the scaffold subunit and CNOT8 is a catalytic deadenylase subunit^31^. Interestingly, for proteins in the CCR4-CAF1-NOT deadenylase complex, we obtained SILAC ratios of ∼1 suggesting that RC3H1 interacts only transiently with these proteins (Supplementary Fig. 3a). Specific interactions of RC3H1 with CNOT1 and CNOT8, but not with the RBP QKI, were confirmed by co-immunoprecipitation experiments in agreement with previous findings^9^ (Fig. 3b), indicating that RC3H1 generally acts as mediator of mRNA deadenylation.

### RC3H1 destabilizes target mRNAs

To assess whether RC3H1 interacts with short-lived mRNA transcripts, we performed transcriptome-wide mRNA half-life measurements (Supplementary Data 3) as described by Dölken *et al.*^32^, and compared half-lives of RC3H1-bound and unbound mRNA transcripts. Consistent with the interaction of RC3H1 with the deadenylase complex and a possible role in mRNA decay, RC3H1-targeted mRNAs were found to have shorter half-lives than expressed non-target transcripts (Supplementary Fig. 3b,c). On the other hand, IGF2BP1-bound transcripts do not show this tendency (Supplementary Fig. 3d,e). Furthermore, we found that mRNA half-lives of RC3H1-bound transcripts inversely correlated with an expression normalized PAR-CLIP score (Supplementary Fig. 3f), suggesting that the extent of RC3H1–mRNA binding determined the mRNA half-lives of bound mRNAs (Supplementary Data 4).

To examine the impact of RC3H1 on the decay rate of its mRNA targets, we sequenced mRNA of untreated and RC3H1- and RC3H2-depleted cells (Supplementary Fig. 3g) after inhibition of transcription using actinomycin D from two biological replicates. Figure 3c indicates that mRNA decay rates of RC3H1-bound transcripts were decreased in cells depleted of RC3H1 and RC3H2, suggesting that RC3H proteins destabilize their mRNA targets.

In addition, we examined the effect of RC3H1 depletion on the protein synthesis rate of RC3H1-bound mRNAs. For this purpose, we monitored changes in newly synthesized proteins by pulsed SILAC (pSILAC) based quantitative proteomics^22^,^33^ upon depletion of endogenous RC3H1 in HEK293 cells (Supplementary Fig.4a). The mass shift between the RC3H1 knockdown (‘medium' labelled) and mock-treated control (‘heavy' labelled) allowed the quantification of changes in protein synthesis of ∼2,400 proteins (Supplementary Fig. 4a). RC3H1 knockdown was confirmed by western blot analysis (Supplementary Fig. 4b). A cumulative distribution function analysis showed that the level of protein synthesis of RC3H1-bound mRNAs was increased upon RC3H1 depletion by two short interfering RNA (siRNAs) (Fig. 3d and Supplementary Fig. 4c,d). Taken together, our data indicate that RC3H1-bound mRNAs are short lived, and depletion of RC3H1 resulted in decreased decay rates and increased proteins synthesis of its target transcripts, validating the functionality of RC3H1–mRNA interactions.

### RC3H1 interacts with DNA damage-induced transcripts

**Figure 4:**
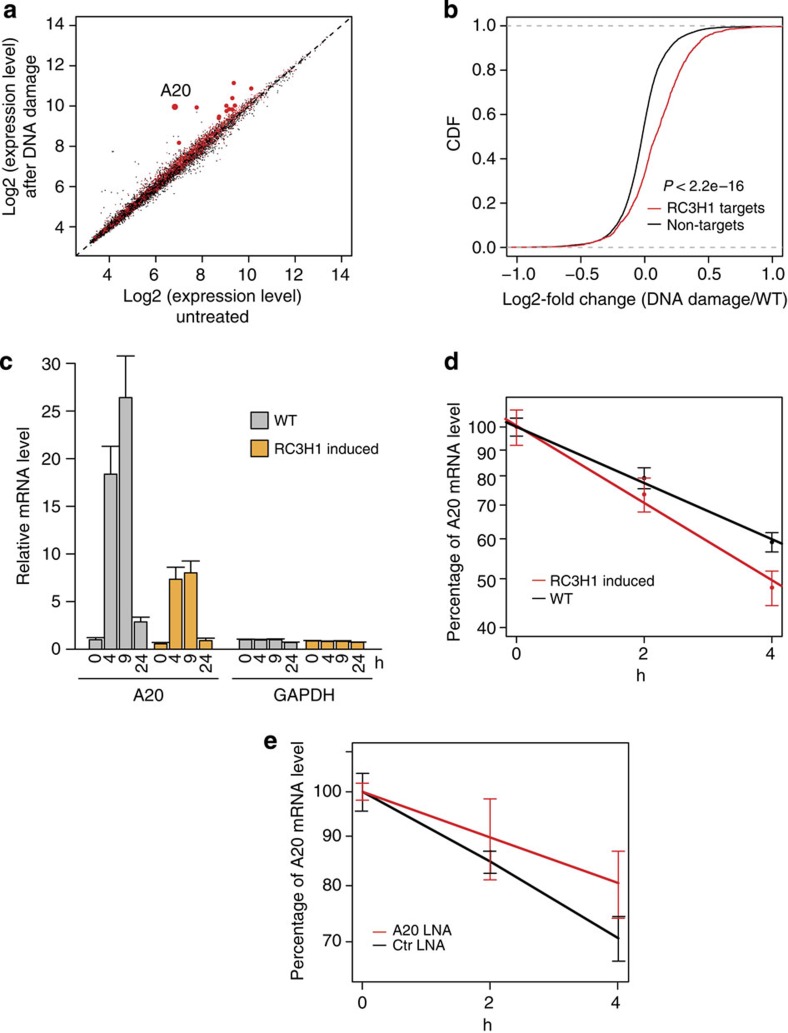
RC3H1 target transcripts are enriched for mRNAs induced upon DNA damage, and RC3H1 negatively regulates A20 at the post-transcriptional level. (**a**) A scatter plot of mRNA expression levels of untreated cells and cells treated for 4 h with 200 ng ml^−1^ of neocarzinostatin (NCS). The data was retrieved from Elkon *et al.*^34^. RC3H1 3′UTR target transcripts are shown in red and non-targets are shown in black. Among the RC3H1 targets, A20 was the most differentially expressed mRNA upon DNA damage. (**b**) A cumulative distribution function (CDF) plot of log2-fold changes upon DNA damage is shown for RC3H1 3′UTR targets in red and for non-targets in black (*P* value <2.2e-16, Wilcoxon-rank sum test). (**c**) RC3H1 induction by doxycycline treatment specifically leads to reduced expression of A20 at each time point. mRNA expression level of A20 and GAPDH (negative control) are measured by qPCR at 0, 4, 9 and 24 h post-DNA damage induced by 250 ng ml^−1^ of NCS. Averages and s.d.'s (error bar) from three technical replicates are shown. A representative data set out of two independent biological replicates is shown. WT, wild type. (**d**) Induction of RC3H1 leads to increased A20 mRNA decay. At 4 h post-DNA damage induced by NCS (250 ng ml^−1^), transcription was blocked with actinomycin D, and mRNA expression levels were measured by qRT–PCR. Percentage of A20 mRNA amount at each time point relative to starting point is shown. Error bars indicate s.d.'s calculated from three replicates. (**e**) Transfection of antisense LNA oligonucleotide targeting the stem–loop structure in HEK293 cells leads to decreased A20 mRNA decay (red) in comparison with control (Ctr) LNA transfection (black). A representative data from two independent experiments are shown.

Our KEGG pathway enrichment analysis revealed that RC3H1 mRNA targets are enriched for genes involved in cell cycle regulation and p53 signalling (Supplementary Fig. 1e) Furthermore, RC3H1 was shown to localize to stress granules upon oxidative stress induced by arsenite exposure^4^, suggesting an involvement of RC3H1 in the cellular stress response. To investigate a possible role of RC3H1 in the DNA damage response, we correlated changes in mRNA expression levels upon DNA damage induced by neocarzinostatin in HEK293 cells^34^ with our RC3H1 PAR-CLIP data (Fig. 4a). Notably, RC3H1-bound transcripts were more induced upon DNA damage than unbound mRNAs (Fig. 4b). This finding implies that the expression levels of DNA damage-induced genes are likely modulated at the post-transcriptional level at least in part by RC3H1. The mRNA of A20, a NF-κB target gene that acts as a feedback regulator of NF-κB activation, was among the RC3H1 target transcripts and showed the largest increase in expression upon DNA damage. Induction of FLAG/HA-tagged RC3H1 (Supplementary Fig. 5a) in HEK293 cells resulted in a significant reduction of A20 mRNA (Fig. 4c) upon DNA damage. Furthermore, we observed that A20 mRNA half-life was shortened by expression of tagged RC3H1 (Fig. 4d). To provide further support for this finding, we specifically blocked the RC3H1-binding site on A20 mRNA by transfecting an antisense locked nucleic acid (LNA) oligonucleotide and observed an increase in A20 mRNA half-life (Fig. 4e).

### RC3H1 binding to A20 3′UTR via ROQ and CCCH-Znf domains

**Figure 5:**
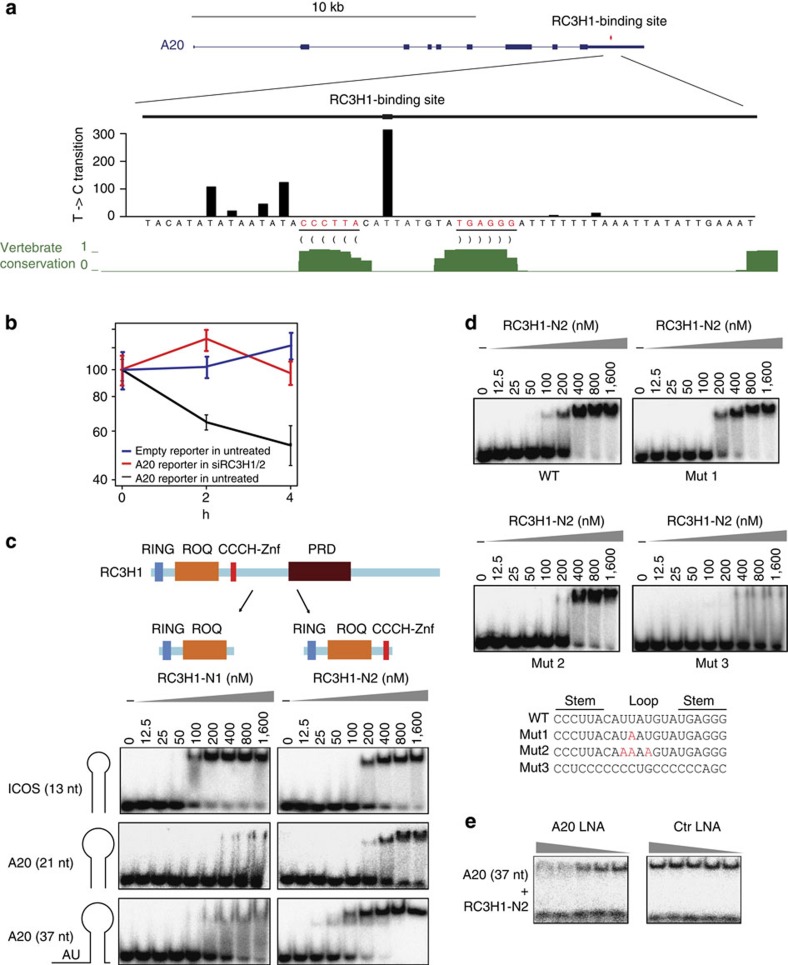
RC3H1 binds to a composite structure-sequence motif in the A20 3′UTR mediated by the CCCH-type Zn-finger domain. (**a**) Illustration of the RC3H1-binding site in the A20 3′UTR. The binding sites of RC3H1 in the 3′UTR of A20 is shown in red and zoomed in below. T to C transitions for indicated base positions are shown. Bases shown in red are forming a potential stem. Phastcon vertebrate conservation is shown in green. RC3H1-binding site in the A20 3′UTR contains a stem–loop structure flanked by AU-rich sequences. (**b**) The effect of A20 AU-rich element (ARE)-stem–loop hairpin (37 nucleotide (nt)) was assayed by transiently transfecting HEK293 cells with the d2GFP reporter plasmid, which contains the 37-nt sequence inserted into the 3′UTR of d2GFP. mRNA decay of the reporter transcripts were measured in mock and RC3H1/RC3H2 knockdown cells. Average and s.d.'s (error bar) from three technical replicates are shown. (**c**) EMSA experiments to examine the binding mode of RC3H1 to the A20 target site. Increasing concentration of recombinant RC3H1-N1 (aa 2–399) or RC3H1-N2 containing an additional CCCH-type Zn-finger domain (aa 2–452) was incubated with radiolabelled ICOS (13 nt), A20 stem–loop (21 nt) and A20 ARE-stem–loop (37 nt), and free RNA was separated from RNA–protein complexes by native PAGE. (**d**) EMSA experiments to examine the sequence specificity of the A20 stem–loop hairpin. Increasing concentration of recombinant RC3H1-N2 was incubated with radiolabelled wild-type (WT) A20 stem–loop (21 nt), mutated A20 sequences (Mut 1 and Mut 2) as indicated below, or 21 nt control sequence (Mut 3) generated by concatenating three 7mers underrepresented in our 7mer analysis. Mutation in the loop slightly reduces the binding, and the control sequence does not virtually bind to RC3H1-N2. (**e**) Increasing concentration of antisense LNA oligonucleotide targeting the A20 stem–loop structure impairs the interaction of RC3H1-N2 and 37 nt ARE-stem–loop.

Our PAR-CLIP data indicated a single RC3H1-binding site in the 3′UTR of A20 mRNA. Diagnostic PAR-CLIP T to C transition events, indicating protein–RNA crosslinking sites, were detected in a loop of a conserved predicted hairpin and in an AU-rich sequence located upstream of the stem–loop structure, which differs from the previously described CDE (Fig. 5a). To examine whether the putative A20-binding site bestows RC3H1-dependent mRNA decay, we cloned a 37-bp sequence covering the crosslinked region into a green fluorescent protein (GFP) reporter and assayed mRNA turnover by quantitative reverse transcription–PCR (qRT–PCR) after blocking transcription using actinomycin D. Indeed, insertion of the RC3H1-bound A20 site into the 3′UTR of the reporter construct destabilized reporter transcripts in mock-transfected cells, but not in RC3H1- and RC3H2-depleted cells (Fig. 5b), indicating that RC3H proteins destabilize the reporter transcripts through this A20 site.

To further examine the RC3H1 interaction with the putative A20 site, we used electrophoretic mobility shift assays (EMSA). In addition, to assess the contribution of the different RC3H1 domains to RNA binding, we expressed two variants: RC3H1-N1 (aa 2–399) contained the N-terminal RING and ROQ domains, whereas RC3H1-N2 (aa 2–452) harboured RING, ROQ and CCCH-type Zn-finger domains (Fig. 5c). Both recombinant proteins bound to the ICOS CDE-like stem–loop motif RNA (Fig. 5c). The formation of the protein–RNA complex seemed to be independent of the CCCH-type Zn-finger domain. In contrast, the 21-nucleotide A20 hairpin RNA was bound by RC3H1-N2 with higher affinity than RC3H1-N1 (Fig. 5c), indicating that the CCCH-type Zn-finger domain plays a role in the interaction with the non-CDE-type A20 site. The addition of 16 nucleotides 5′ of the stem–loop structure (A20 37 nucleotide) further increased the affinity of the RC3H1-N2 variant to the RNA substrate (Fig. 5c), suggesting that an additional sequence upstream of the A20 hairpin is involved in protein–RNA complex formation. To further study the specificity of RC3H1 interaction to its A20 stem–loop hairpin, we performed EMSA using stem–loop hairpin or variants thereof (Fig. 5d). A single-nucleotide substitution in the loop region virtually did not affect binding; however, substitution of three nucleotides resulted in slight reduction of binding (Fig. 5d). In contrast, a control sequence, which was generated by concatenating three 7mers underrepresented in our 7mer analysis, did not bind to RC3H1, indicating a specificity for the A20 hairpin (Fig. 5d). Moreover, the antisense LNA oligonucleotide, which was used to modulate A20 mRNA stability (Fig. 4e) and hybridizes to the loop and the 3′part of the stem, reduced the binding of RC3H1-N2 to the 37-nucleotide A20 target sequence, suggesting that the specific blockade of both loop and stem pronouncedly reduced RC3H1 binding to A20 3′UTR (Fig. 5e).

### RC3H1 represses A20 and modulates the NF-κB pathway

**Figure 6:**
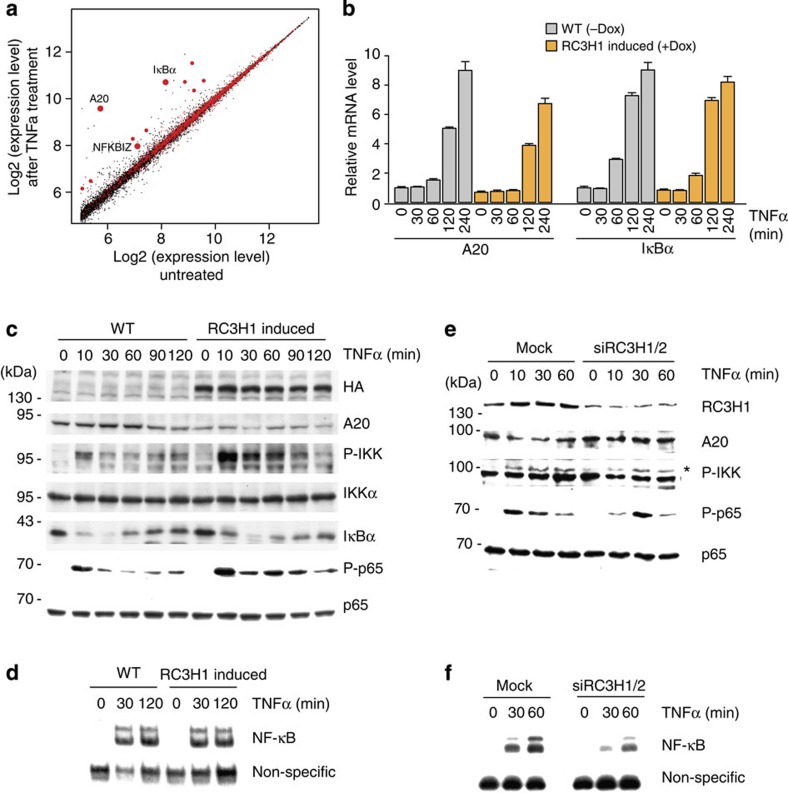
RC3H1 modulates the activation of IKK by TNFα. (**a**) A scatter plot of mRNA expression levels of untreated cells and cells treated for 4 h with 10 ng ml^−1^ of TNFα. The data were retrieved from Grimley *et al.*^37^. RC3H1 3′UTR target transcripts are shown in red and non-targets are shown in black. Several TNFα-induced mRNAs, such as A20, IκBα and NFKBIZ, are targets of RC3H1. Among the RC3H1 target transcripts, A20 was the most differentially expressed mRNA upon TNFα treatment. (**b**) RC3H1 induction leads to slightly reduced expression of A20 and IκBα at each time point. mRNA expression levels of A20 (left) and IκBα (right) were measured by qPCR at indicated time points after TNFα treatment. Representative data from two independent experiments are shown. Average and s.e.m. (error bar) are from three technical replicates. (**c**) Western blot analyses of the NF-κB pathway proteins after TNFα stimulation in cells with doxycyline (Dox)-dependent RC3H1 expression. HEK293 cells were treated with Dox (1 μg/ml for 72 h), to induce HA-RC3H1. Subsequently, cells were treated with TNFα as indicated, and analysed by western blot with the indicated antibodies. RC3H1 upregulation results in decreased A20 expression, leading to increased IKK activation (T-loop phosphorylation, P-IKK) and phosphorylation of p65 (P-p65). Representative data from two independent experiments are shown. (**d**) EMSA analysis of whole-cell extracts for TNFα-induced NF-κB activity. Cells were treated as in **c**. (**e**) Western blot analyses of the NF-κB pathway proteins after TNFα stimulation in mock or RC3H1/2 siRNA-treated HEK293. Cells were treated with TNFα, and analysed by western blot with the indicated antibodies. RC3H1 downregulation results in mildly increased A20 expression, leading to decreased IKK activation and phosphorylation of p65. Representative data from two independent experiments are shown. ‘*' indicates phosphorylated form of IKK. (**f**) EMSA analysis of whole-cell extracts for TNFα-induced NF-κB activity. Cells were treated as in **e**. Knockdown of RC3H1 expression reduced the NF-κB activity.

Notably, RC3H1-dependent mRNA regulation affects several NF-κB pathway regulators^5^,^9^. A recent transcriptome-wide RIP-seq study determining RC3H1-bound transcripts in mouse macrophages revealed the cytokine TNFα—a typical activator of NF-κB^35^,^36^—and two members of the IκB family, IκBNS (NFKBID) and IκB-zeta (NFKBIZ)^9^. In addition to the ubiquitin-editing enzyme A20, we identified IκBα in our PAR-CLIP as an additional RC3H1 target transcript (Fig. 6a,b). To examine whether RC3H1 acts in a pathway-specific manner, we used previously published mRNA expression data of TNFα-treated HEK 293 cells^37^ to correlate RC3H1-bound mRNAs with TNFα-induced transcripts. Interestingly, RC3H1 target transcripts showed a greater increase in expression upon TNFα induction than non-targets, suggesting that RC3H1 acts post-transcriptionally on NF-κB target genes (Fig. 6a and Supplementary Fig. 5b). Furthermore, RC3H1 induction caused a reduction of basal and stimulus-dependent A20 mRNA and protein levels (Fig. 6b,c), similar to A20 expression changes observed during the response after DNA damage (Fig. 4c).

Next, we asked whether RC3H1 could modulate NF-κB activity. NF-κB activation is mediated via the IKK complex, which catalyses the phosphorylation of IκB and NF-κB proteins, as well as of other substrates^35^,^36^,^38^,^39^. Signalling involves ubiquitin-mediated complex formation of pathway components and is controlled at various levels by negative feedback mechanisms, including ubiquitin-editing enzymes such as A20 (ref. 40).

Indeed, RC3H1-mediated decrease of A20 protein levels led to a significant increase of IKK activation (Fig. 6c), which was repressed by additionally expressing exogenous A20 without 3′UTR (Supplementary Fig. 5c). In line with this, elevated Ser536 phosphorylation of the IKK substrate p65 was observed (Fig. 6c). However, IκBα degradation and NF-κB DNA-binding activity were not detectably affected (Fig. 6c,d). To investigate the impact of RC3H1 ectopic expression on the kinetics of the NF-κB pathway and to understand the differential effect on IKK versus NF-κB, a mathematical modelling approach was used, including main processes of canonical IKK/NF-κB signalling (Supplementary Fig. 5d). The model parameters were estimated based on the western blot analysis for A20, IκBα and phosphorylated IKK (Fig. 6c), as well as qPCR data for IκBα and A20 mRNA levels (Fig. 6b; for details see Methods section). The model simulations (Supplementary Fig. 5d) confirmed the experimental findings that RC3H1 reduces A20 mRNA and subsequently A20 protein expression, and, due to the attenuated IKK inhibition by A20, leads to an increased IKK activity. In turn, NF-κB activity is slightly increased by RC3H1, resulting in an increased mRNA synthesis of the feedback regulators IκBα and A20. According to the model, enhanced mRNA syntheses are counteracted by the increased mRNA decay mediated by RC3H1 induction. For IκBα, this establishes a compensatory mechanism resulting in largely unaffected IκBα mRNA and protein levels upon ectopic RC3H1 expression (Fig. 6b,c).

In contrast, knockdown of RC3H1 and RC3H2 in HEK293 cells resulted in a small, but reproducible, upregulation of A20 protein expression (Fig. 6e), which resulted in decreased phosphorylation of IKK (Fig. 6e), decreased phosphorylation of its substrate p65 (Fig. 6e) and reduced NF-κB DNA-binding activity (Fig. 6f). These observations are also reproduced by the mathematical model (Supplementary Fig. 5d) showing that a strengthening of the A20 feedback leads to changes in activated levels of IKK and NF-κB.

Taken together, we could demonstrate that RC3H1 regulates the expression of several NF-κB pathway regulators, thereby modulating IKK and NF-κB activity.

## Discussion

In the present study, we identified transcriptome-wide RNA-binding sites of human RC3H1 at nucleotide resolution in HEK293 cells using PAR-CLIP. Our bioinformatic analyses did not reveal a well-defined motif as observed for sets of RBPs^41^; however, indicated several classes of sequence-structure binding elements with U-rich sequences frequently embedded in RNA stem–loop structures in 3′UTRs of target transcripts. Surprisingly, the CDE core consensus motif (UCYRYGA) deduced by Leppek *et al.*^9^ was present only in a minor fraction of identified RC3H1-binding sites. Our RC3H1 PAR-CLIP data are also in agreement with the concept of a relaxed CDE revealed by structural and mutational analyses^10^, which indicated a shape-specific rather than sequence-specific recognition of CDE hairpins by the ROQ domain.

Interestingly, our finding of a PAR-CLIP cluster in the A20 3′UTR indicated a yet unrecognized RC3H1-binding mode and specificity. In contrast to a typical CDE stem–loop motif, which is sufficiently bound by the ROQ domain, we provide evidence that the CCCH-type Zn-finger domain is involved in contacting the A20 site. A RC3H1 variant containing the CCCH-type Zn-finger domain bound with higher affinity to a non-CDE-like stem–loop structure with an additional AU-rich sequence upstream of the hairpin than to the hairpin alone. In contrast, the N-terminal RC3H1 variant lacking the CCCH-type Zn-finger domain poorly bound to both of these RNA substrates. The makeup of RC3H1 by distinct RNA-binding domains might allow the protein to recognize a wider range of RNA structure-sequence elements and could function on a larger set of regulatory elements than previously anticipated. The ratio of sequence and structure specificity features, determining the strength of the RC3H1–mRNA association, and the RNA recognition element frequency would influence the regulatory capacity of the RBP.

In addition, our results indicate that RC3H1 interacts with the CCR4-CAF1-NOT deadenylation complex, and mediates destabilization of RC3H1 target transcripts. RC3H1-bound mRNAs are encoded by genes with various biological functions outside of immune-response pathways, which is in accordance with the mouse phenotype of *Rc3h1 null*-knockout that showed perinatal lethality with broad physiological complications^42^. Enriched KEGG pathways included cell cycle, p53 signalling and tumour pathways. By intersecting our PAR-CLIP target mRNAs with mRNA expression data, we found that RC3H1 targets are enriched for mRNAs induced by DNA damage^34^ and TNFα^37^. As shown for one of the top mRNA targets, A20, we postulate that RC3H1, in general, is involved in fine-tuning or clearance of transcriptionally induced mRNAs by shortening their half-lives.

Our discovery of RC3H1 binding to A20 mRNA and other TNFα-induced transcripts prompted us to examine and model the impact of RC3H1 on the IKK/NF-κB pathway. Knockdown of RC3H1 and RC3H2 increased the expression of A20 protein expression, resulting in reduced IKK activity and NF-κB DNA-binding activity. Vice versa, we show that induction of RC3H1 results in pronounced increase of IKK phosphorylation. Taken together, RC3H1 targets several components of the NF-κB signalling pathway, and thereby modulates IKK and NF-κB activity. The net impact of alterations in RC3H protein activity on IKK and NF-κB activity in different cell types most likely depends on various additional (cell-type) specific parameters. Notably, IKK does not only regulate NF-κB activation but is also engaged in crosstalk with other pathways^40^.

The Zn-finger protein A20 is an important negative regulator of inflammation^21^, and several studies have highlighted the clinical and biological importance of A20. Walle *et al.*^43^ recently showed that negative regulation of the NLRP3 inflammasome by A20 protects against arthritis. Since RC3H1 is a negative regulator of A20, targeting of the RC3H1-A20 mRNA interaction by using antisense technologies and concomitant upregulation of A20 protein might have beneficial outcomes in certain disease scenarios.

In summary, we identified comprehensive RC3H1-binding sites by PAR-CLIP, revealing a large number of novel mRNA targets as well as novel RC3H1 *cis*-acting recognition element in the A20 3′UTR. Our study highlights the importance of post-transcriptional regulation of gene expression to control crucial cellular signal transduction pathways.

## Methods

### Antibodies

For western blots, the following antibodies were used after dilution to 0.5–1 μg ml^−1^: anti-HA.11 (Covance, 16B12), anti-FLAG (Sigma, F1804), anti-myc (Sigma, 9E10) anti-γH2AX(Upstate, JBW301), anti-vinculin (Sigma, hVIN-1), anti-RC3H1 (Novus, NB100–655), anti-A20 (Santa Cruz Biotechnology, sc-32525), anti-pIKK (Cell Signal Technology, 2,697), anti-IKKα (BD Pharmingen, 5,56,532), anti-IκBα (Santa Cruz Biotechnology, sc-371), anti-p65 (Santa Cruz Biotechnology, sc-8008P), anti-p-p65 (Cell Signaling Technology, 3033) and polyclonal goat anti-mouse or anti-rabbit immunoglobulins/horseradish peroxidase (Dako).

### Oligonucleotides

#### siRNAs

siRNA 1 for RC3H1: 5′-GCUGGGAAAUACAAAGGAA[dT][dT].

siRNA 2 for RC3H1: 5′-CCAAGAAAUGUGUAGAAGA[dT][dT].

RC3H2: 5′-GGAAGAAGGUCGUGUAAGA[dT][dT].

#### qPCR primers

RC3H1 forward: 5′-tggacaaccagaaccacaaa-3′; reverse: 5′-GCTGATCCATTTGGTACATCAC-3′.

A20 forward: 5′-TGCACACTGTGTTTCATCGAG-3′; reverse: 5′-ACGCTGTGGGACTGACTTTC-3′.

RPL18A forward: 5′-GGAGAGCACGCCATGAAG; reverse: 5′-AAGATTCGCATGCGGTAGAG-3′.

GAPDH forward: 5′-AGCCACATCGCTCAGACAC-3′; reverse: 5′-GCCCAATACGACCAAATCC-3′.

NFKBIA forward: 5′-GAGTCAGAGTTCACGGAGTTC-3′; reverse: 5′-CATGTTCTTTCAGCCCCTTTG-3′.

d2GFP forward: 5′-GAAGCTTAGCCATGGCTTCCC-3′; reverse: 5′-GATGGCCGCATCTACACATTG-3′.

#### DNA oligos for d2GFP-A20 3′UTR reporter

Sense: 5′-GGCCTGTACATATATAATATACCCTTACATTATGTATGAGGGATTTT-3′; antisense: 5′-TCGAAAAATCCCTCATACATAATGTAAGGGTATATTATATATGTACA-3′.

#### RNA oligos

ICOS (13 nucleotide): 5′-AUUUCUGUGAAAU-3′.

A20 (21 nucleotide): 5′-CCCUUACAUUAUGUAUGAGGG-3′.

A20 (37 nucleotide): 5′-AUAUAUAAUAUACCCUUACAUUAUGUAUGAGGGAUUU-3′.

Mut1 (21 nucleotide): 5′-CCCUUACAUAAUGUAUGAGGG-3′.

Mut2 (21 nucleotide): 5′-CCCUUACAAAAAGUAUGAGGG-3′.

Mut3 (21 nucleotide): 5′-CCUCCCCCCCUGCCCCCCAGC-3′.

### Plasmids

pENTR4 constructs were generated by PCR amplification of the RC3H1 and QKI5 coding sequences from cDNA followed by restriction digest and ligation into the pENTR4 (Invitrogen) backbone, which were further recombined into the pFRT/TO/FLAG/HA-DEST destination vector^44^ using GATEWAY LR recombinase (Invitrogen) according to manufacturer's protocol. Expression plasmids for HA-tagged CNOT1 and CNOT8 were kind gifts from Dr W. Filipowicz. pENTR4 QKI5 was recombined into pFRT/FLAG/HA-DEST (Addgene ID: 26,360). The d2GFP reporter plasmids were generated by cloning the d2GFP (Clontech) coding sequence into pcDNA5/FRT, and synthetic DNA oligonucleotides containing the A20-binding site were annealed and ligated into the 3′UTR of d2GFP using the Xho1/Not1 site.

### Cell lines and culture conditions

Flp-In 293 T-REx cells (Invitrogen) were grown in Dulbecco's modified Eagle's medium high glucose with 10% (v/v) fetal bovine serum, 2 mM L-glutamine. Cell lines stably expressing FLAG/HA-tagged RC3H1 protein were generated by co-transfection of pFRT/TO/FLAG/HA constructs with pOG44 (Invitrogen). Cells were selected by adding 15 μg ml^−1^ blasticidin and 100 μg ml^−1^ hygromycin (Invivogen). Expression of epitope-tagged proteins was induced by addition of 1 μg ml^−1^ doxycyclin. The expression of FLAG/HA-tagged RC3H1 protein was assessed by western analysis using mouse anti-HA.11 monoclonal antibody (Covance). For quantitative proteomics, cells were grown in SILAC medium as described before^30^,^45^. Briefly, Dulbecco's modified Eagle's medium GlutaMAX lacking arginine and lysine (PAA) supplemented with 10% dialysed fetal bovine serum (Gibco) was used. Amino acids (84 mg l^−1 13^C_6_^15^N_4_ L-arginine plus 146 mg l^−1 13^C_6_^15^N_2_ L-lysine or 84 mg l^-1 13^C_6_-L-arginine plus 146 mg l^−1^ D4-L-lysine) or the corresponding non-labelled amino acids (Sigma) were added to obtain ‘heavy' ‘medium' or ‘light' cell culture medium, respectively. Labelled amino acids were purchased from Sigma Isotec.

### Western blot analysis

Total cell lysates were prepared in 1 × SDS–PAGE sample loading buffer (50 mM Tris (pH 7.5), mercaptoethanol, 1% SDS, 0.01% bromophenol blue and 10% glycerol) and resolved by SDS–PAGE. Proteins were transferred to nitrocellulose membrane (Whatman) using a semi-dry blotting apparatus (Bio-Rad) at constant 20 V for 1 h. The membrane was blocked in 5% non-fat milk and incubated with primary antibody. Following incubation for 1 h at room temperature, membranes were washed three times in TBST (150 mM NaCl, 20 mM Tris-HCl (pH 7.5) and 0.1 % Tween) and incubated with horseradish peroxidase-conjugated secondary antibody for 1 h. Following three additional TBST washes, protein bands were visualized using ECL detection reagent (GE Healthcare) and a LAS-4000 imaging system (GE Healthcare). Uncropped images of data shown in the figures are provided in Supplementary Fig. 6.

### PAR-CLIP

Stably transfected and inducible FLAG/HA-RC3H1 expressing cells were labelled with 100 μM 4SU or 6SG for 8–9 h. After labelling the cells, PAR-CLIP was performed essentially as described in ref. 19. Briefly, for 4SU-2 and one 6SG, ultraviolet-irradiated cells were lysed in NP40 lysis buffer (50 mM HEPES-KOH at pH 7.4, 150 mM KCl, 2 mM EDTA, 0.5% (v/v) NP40, 0.5 mM dithiothreitol (DTT) and complete EDTA-free protease inhibitor cocktail). After mild treatment with RNase T1 (Fermentas) at final concentration of 1 U μl^−1^ for 15 min at room temperature, immunoprecipitation was carried out with protein-G magnetic beads (Invitrogen) coupled to anti-FLAG M2 antibody (Sigma) from extracts of FLAG/HA-RC3H1 expressing and 4SU-labelled HEK 293 cells for 1 h at 4 °C. For 4SU-1, a high-salt lysis buffer (50 mM Tris-HCl, 500 mM NaCl, 1% (w/v) NP40, 1 mM DTT and complete EDTA-free protease inhibitor cocktail) was used for cell lysis followed by sonication. After mild treatment with RNaseT1 at final concentration of 1 unit per μl for 15 min at room temperature, purification of the RC3H1/RNA complex was performed with Flag magnetic beads (Sigma). Following additional digestion by RNase T1 (Fermentas) at final concentration of 10 unit per μl for 2 min at room temperature, beads were incubated with calf intestinal phosphatase (NEB) and RNA fragments were radioactively end labelled using T4 polynucleotide kinase (Fermentas). The crosslinked protein–RNA complexes were resolved on a 4–12% NuPAGE gel (Invitrogen). The SDS–PAGE gel was transferred to a nitrocellulose membrane (Whatman) and the protein–RNA complex migrating at an expected molecular weight was excised. RNA was isolated by proteinase K (Roche) treatment and phenol–chloroform extraction, ligated to 3′ adapter. (5′-AppTCGTATGCCGTCTTCTGCTTG-InvdT-3′ for 4SU-1, 5′-AppTCTCGTATCGTATGCCGTCTTCTGCTTG-InvdT-3′ for 4SU-2 and 5′-AppTCTCTGCTCGTATGCCGTCTTCTGCTTG-InvdT-3′ for 6SG), and 5′ adapter (5′-rGrUrUrCrArGrArGrUrUrCrUrArCrArGrUrCrCrGrArCrGrArUrC-3′), reverse transcribed and PCR amplified. The amplified cDNA was sequenced on a HighSeq2000 (Illumina) with a 1 × 51 nucleotide cycle for 4SU-2 and 6SG and on a Genome Analyzer II with a 1 × 36 cycle for 4SU-1.

### PAR-CLIP data processing

The PAR-CLIP cDNA sequencing data were processed by an automated PAR-CLIP analysis pipeline developed by Lebedeva *et al.*^22^ with default settings. Briefly, the PAR-CLIP analysis pipeline performs the following steps: (1) reads were mapped to the genome, and uniquely aligned reads with up to one mismatch, insertion or deletion were used to build binding clusters; (2) each binding cluster was assigned a score based on the number of T to C or G to A mismatches and on the heterogeneity of distinct reads contributing to the cluster. As the reads should originate from RC3H1-bound transcripts, we regarded clusters aligning antisense to the annotated direction of transcription as false positives. (3) Filtering was performed to obtain RC3H1 clusters sites at an estimated 5% false-positive rate based on the assigned score for each cluster. For each cluster, the position with the highest number of diagnostic transition events was determined, and we defined this position as the preferred crosslink site. To define the consensus clusters, we pooled reads from all three experiments while ensuring that transition events are counted appropriately (T to C only in reads originating from 4SU experiments and G to A only in reads from the 6SG experiment). Before the cutoff determination, clusters had to pass an additional consensus filter, demanding that reads from at least two out of the three experiments support the cluster. The resulting sets of clusters were denoted as the ‘consensus' set. Read alignment statistics, cluster length distribution, target gene identification, cluster distribution, cluster coverage profiles, conservation profile and miRNA target scan were generated by the PAR-CLIP analysis pipeline. The IGF2BP1 PAR-CLIP data were obtained from GEO (GSE21578; ref. 19) and analysed using the same PAR-CLIP analysis pipeline with similar settings. KEGG pathway and GO term enrichment analysis was performed using the on-line DAVID programme^46^,^47^. The top 1,000 transcripts (ranked by the number of PAR-CLIP diagnostic mutations falling into 3′UTR) were used for pathway enrichment analysis.

### Motif analysis

7mer occurrences were counted in 41 nucleotide windows around the crosslink site identified in the 4SU and 6SG PAR-CLIP experiments using custom Perl scripts. To examine the enrichment of each 7mer motif, 7mer frequency occurring in RC3H1 consensus 3′UTR-binding sites was compared with that occurring in all 3′UTR sequences retrieved from UTRdb^48^. The longest 3′UTR sequence for each gene was used in this analysis. To test whether RC3H1-binding sites showed a preferred secondary structure, we used the library routines from the Vienna RNA package 1.8.2 (ref. 49) to compute base pairing probabilities within 41 nucleotide sequences centred on the preferred crosslink positions of 3′UTR-binding sites. The resulting profiles were accumulated and averaged over all 3′UTR consensus binding sites or the negative control 41 nucleotide sequences randomly selected from the 3′UTRs of RC3H1 target transcripts. Stem–loop enrichment analysis was done based on the output from RNAfold programme in the Vienna RNA package. For the clustering of structured motifs, we started with the top 100 (ranked by number of diagnostic transition events divided by expression value) RC3H1-binding sites (41 nucleotide length sequence centred around the preferred crosslink site) determined by PAR-CLIP, and detected initial clusters by LocARNA (version 1.7.16; refs 26, 27) together with RNAclust (version 1.3; ref. 27) to produce a hierarchical tree. RNAclust (which uses LocARNA) was run using default parameters together with the RNAsoup option (./RNAclust.pl –fasta top100_sequences.fasta –dir output_dir/ --RNAsoup). For each subcluster, we used CMfinder (version 0.2; ref. 28) to search for a subset of sequences that has a conserved sequence-structure motif. CMfinder generates both a sequence-structure alignment (called seed alignment) as well as a covariance model, which we used to search for further sequences in the top 1,000 binding sites for remote members of this motif using cmsearch from the Infernal package (version 1.0.7; ref. 29). We then cut the hierarchical tree at the point where we got structured motifs with the largest coverage in the list of top 1,000 binding sites, which resulted in three structured motifs. We kept two motifs (motifs 1 and 2) and discarded the third one, since its seed alignment consisted of only five entries.

### siRNA knockdown and pSILAC

Flp-In 293 T-REx cells were grown in SILAC medium supplemented with ‘light' labelled amino acids before siRNA knockdown experiments. siRNAs were transfected at a final concentration of 50 nM using Lipofectamine RNAiMAX (Invitrogen). Controls (mock) were treated with transfection reagent only. Following 24 h of incubation, siRNA-transfected cells were switched to ‘medium' labelled SILAC medium, whereas mock control cells were switched to ‘heavy' labelled SILAC medium. After 24 h of labelling, cells were harvested and equal amounts of siRNA- and mock-transfected cells were pooled, lysed in urea buffer (8 M urea and 100 mM Tris-HCl, pH 8.3) and sonicated for 20 s (two pulses, 60% power). Cell debris was removed by centrifugation (14,000*g*, 5 min). Protein concentration was then measured by the Bradford colorimetric assay. An amount of 100 μg of proteins were reduced in 2 mM DTT for 30 min at 25 °C, and successively free cysteines were alkylated in 11 mM iodoacetamide for 20 min at room temperature in the dark. LysC digestion was performed by adding LysC (Wako) in a ratio 1:40 (w/w) to the sample and incubating it for 18 h under gentle shaking at 30 °C. After LysC digestion, the samples were diluted three times with 50 mM ammonium bicarbonate solution, 7 μl of immobilized trypsin (Applied Biosystems) were added and samples were incubated 4 h under rotation at 30 °C. Digestion was stopped by acidification with 10 μl of trifluoroacetic acid and trypsin beads were removed by centrifugation. Fifteen micrograms of digest were desalted on STAGE Tips, dried and reconstituted to 20 μl of 0.5 % acetic acid in water^50^. A volume of 5 μl of each sample were injected in duplicate on a Liquid chromatography-tandem mass spectrometry (LS-MS/MS) system (nanoLC-Ultra 1D (Eksigent) coupled to LTQ-Orbitrap Velos (Thermo)), using a 240-min gradient ranging from 5 to 40% of solvent B (80% acetonitrile, 0.1 % formic acid; solvent A=5% acetonitrile and 0.1 % formic acid). For the chromatographic separation, ∼25-cm-long capillary (75 μm inner diameter) was packed with 1.8 μm C18 beads (Reprosil-AQ, Dr Maisch). The capillary nanospray tip was generated using a laser puller (P-2000 Laser Based Micropipette Puller, Sutter Instruments), allowing fritless packing. The nanospray source was operated with spay voltage of 2.1 kV and ion transfer tube temperature of 260 °C. Data were acquired in data-dependent mode, with one survey mass spectrometry (MS) scan in the Orbitrap mass analyser (resolution 60,000 at *m/z* 400) followed by up to 20 MS/MS in the ion trap on the most intense ions (intensity threshold=750 counts). Once selected for fragmentation, ions were excluded from further selection for 30 s in order to increase new sequencing events. Raw data were analysed using the MaxQuant proteomics pipeline (v1.3.0.5) and the built-in Andromeda search engine^51^ with the International Protein Index Human version 3.71 database. Carbamidomethylation of cysteines was chosen as fixed modification, and oxidation of methionine and acetylation of N terminus were chosen as variable modifications. The search engine peptide assignments were filtered at 1% False Discovery Rate (FDR) and the feature match between runs was enabled; other parameters were left as default. For SILAC analysis, two ratio counts were set as threshold for quantification

### RC3H1 protein interactome

For the identification of proteins directly interacting with RC3H1, cells are grown in medium supplemented with either light or heavy stable isotope-labelled amino acids. In the forward experiments, FLAG/HA-tagged RC3H1 was expressed only in cells cultured in light medium, and in the reverse experiments the labelling was swapped. Equal amounts of cells were mixed and lysed in three pellet volumes of NP40 lysis buffer (50 mM Tris-HCl (pH 7.5), 150 mM KCl, 2 mM EDTA (pH 8.0), 0.5% NP40, 1 mM NaF, 0.5 mM DTT and protease inhibitor cocktail). The extracts were treated with 1 unit per μl RNase T1 for 5 min at 22 °C to facilitate the immunoprecipitation and incubated with FLAG magnetic beads (Sigma; 50 μl 1^−1^ ml cell lysate) for 1 h at 4 °C. Beads were washed once with NP40 lysis buffer and treated with 50 unit per μl RNase T1 and 0.25 unit per μl RNase I for 5 min at 37 °C to disrupt RNA-mediated protein interactions. After washing the beads once with FLAG elution buffer (100 mM NaCl, 20 mM Tris-HCl (pH 7.5), 5 mM MgCl_2_ and 10% glycerol), FLAG/HA-RC3H1 complex was eluted by adding 0.5 μg ml^−1^ FLAG peptide and rotating for 1 h at 4 °C. μMACS HA magnetic beads (50 μl per ml cell lysate) was added to FLAG eluate and incubated on ice for 30 min. FLAG eluate anti-HA bead mix was added to the μMACS column after equilibrating with FLAG elution buffer. After washing the column three times with 800 μl ice-cold wash buffer I (150 mM NaCl, 50 mM Tris-HCl (pH 7.5), 5% glycerol and 0.05% NP40) and twice with 500 μl ice-cold wash buffer II (50 mM NaCl, 50 mM Tris-HCl (pH 7.5) and 5% glycerol), a tryptic digestion was performed on-column by adding 25 μl 2 M urea in 100 mM Tris-HCl (pH 7.5), 1 mM DTT and 150 ng trypsin (Promega). After in-column digestion for 30 min at room temperature, peptides were eluted by adding two times 50 μl 2 M urea in 100 mM Tris-HCl (pH 7.5) and 5 mM iodoacetamide. Proteins were further digested overnight at room temperature being protected from light, and digestion was stopped by adding 1 μl trifluoroacetic acid. Resulting peptides were analysed by mass spectrometry.

### Quantitative PCR

Cells were harvested and total RNA was isolated using Trizol (Invitrogen) according to manufacturer's protocol. Total RNA was treated with DNaseI (Invitrogen), and cDNA synthesis was performed using Superscript III (Invitrogen) with oligo-dT primer (18–20 nucleotides) or random hexamer primer (Invitrogen) according to manufacturer's protocol. qPCR analysis was performed with Power SYBR Green PCR Master Mix (ABI) and ABI light cycler as described in the manufacturer's instructions.

### mRNA decay measurement by qRT–PCR

Cells were treated with 5 μg ml^−1^ of actinomycin D (Sigma-Aldrich) to block the transcription. At 0, 2 and 4 h post-actinomycin D treatment, total RNA was harvested using Trizol (Invitrogen) according to manufacturer's protocol. Abundance of specific RNA was quantified by qRT–PCR. mRNA levels were normalized against RPL18A mRNA and plotted against time.

### LNA transfection

LNA oligonucleotide (Exiqon) antisense to the RC3H1-bound stem–loop located in the 3′UTR of A20 (+AA+AT+CC+CT+CA+TA+CA+TAA+T) was transfected at a final concentration of 100 nM using Lipofectamine RNAiMAX (Invitrogen). For control experiment, control LNA (Exiqon) targeting a region in the 3′UTR of A20 (+TCCA+CCTC+CCCT+CCC+CC+A) not bound by RC3H1 was transfected as above. Note that + indicates that the following position is a LNA-modified residue. For mRNA decay assay after antisense inhibition at 4 h after the transfection of LNA, medium was replaced with fresh medium containing 250 ng ml^−1^ of neocarzinostatin (Sigma-Aldrich) to induce DNA damage and A20 expression. 5 h after induction of DNA damage, mRNA decay assay was performed. Random hexamer primers (Invitrogen) were used for cDNA generation.

### mRNA half-life and decay measurements

To calculate the changes of mRNA decay rates upon depletion of RC3H1 and RC3H2, cells were transfected with RC3H1/RC3H2 siRNAs at days 0 and 3. At day 6, untreated and siRNA-treated cells were supplemented with actinomycin D (50 μg ml^−1^) to block transcription, and harvested at time 0, 1 and 2 h after actinomycin D treatment. Experiments are done in biological replicates for each time point. Total RNA was extracted using Trizol, supplemented with 5% *Drosophila melanogaster* total RNA and subjected to high-throughput sequencing using the TruSeq RNA sample prep v2 kit (Illumina). The 12 samples were sequenced on a Illumina HiSeq 2,500 and mapped to the human genome version hg18 using tophat2 (ref. 52). Non-redundant reads per gene were counted using quasR^53^. For decay determination, read counts were divided by the sum of the reads matching to the *Drosophila* transcriptome and by transcript length, and log2-transformed. Decay rates were estimated using linear least-squares regression on the data of two biological replicates. We used quantile normalization of the two sets of decay rates to remove potential biases from normalization. Differences in decay rates were computed by subtracting decay rates of siRNA-treated samples from the decay rates of the mock-treated samples. 4SU-based measurement of mRNA half-lives was performed as described in ref. 32. Briefly, HEK293 cells were treated with 100 μM 4SU for 60 min. Total cellular RNA was isolated using Trizol reagent. Biotinylation of 4SU-labelled RNA was performed using EZ-Link Biotin-HPDP (N-[6-(biotinamido)hexyl]-3′-(2′-pyridyldithio)propionamide) (Pierce) dissolved in dimethylformamide. Biotinylation was carried out in 10 mM Tris (pH 7.4), 1 mM EDTA and 0.2 mg ml^−1^ biotin-HPDP at a final RNA concentration of 100 ng μl^−1^ for 1.5 h at room temperature. An amount of 50–100 μg of total RNA was used for the biotinylation reaction. Unbound Biotin-HPDP was efficiently removed by chloroform:isoamyl alcohol (24:1) extraction using Phase-lock-gel (heavy) tubes (Eppendorf). Then, a 1/10 volume of 5 M NaCl and an equal volume of isopropanol were added, and RNA was precipitated at 20,000*g* for 20 min. The pellet was washed with an equal volume of 75% ethanol and precipitated at 20,000*g* for 10 min. The pellet was resuspended in 50–100 μl RNase-free water. After denaturation of RNA samples at 65 °C for 10 min followed by rapid cooling on ice for 5 min, biotinylated RNA was captured using μMACS streptavidin beads and columns (Miltenyi). Up to 100 μg of biotinylated RNA were incubated with 100 μl of μMACS streptavidin beads with rotation for 15 min at room temperature. The beads were transferred and magnetically fixed to the columns. Columns were washed three times with 1 ml 65 °C washing buffer (100 mM Tris-HCl (pH 7.4) 10 mM EDTA, 1 M NaCl and 0.1% Tween20) followed by three washes with room temperature washing buffer. To recover the unlabelled pre-existing RNA the flow-through of the first two washes was collected and combined. Labelled RNA was eluted by adding 100 μl of freshly prepared 100 mM DTT followed by a second elution 5 min later. RNA was recovered from the washing fractions and eluates using the RNeasy MinElute Spin columns (Qiagen). Total RNA (1.5 μg) and newly transcribed RNA (280 ng) were amplified and labelled using the Affymetrix One-Cycle Target Labeling Kit according to the manufacturer's protocol. As newly transcribed RNA mainly consists of mRNA, it was amplified and labelled according to the manufacturer's protocol for mRNA. The amplified and fragmented biotinylated cRNA was hybridized to Affymetrix Human Gene 1.0 ST Arrays using standard procedures. Data were processed and analysed with R and Bioconductor. To calculate RNA half-lives, CEL-files of all samples from all conditions (including total RNA, newly transcribed RNA and pre-existing RNA) were normalized together using the GCRMA algorithm. Only probe sets called ‘present' in all three replicates of all three RNA subsets under study were included in the analysis of transcript half-lives. Calculation of RNA half-lives was done as performed 32. Statistical comparison of half-life values between groups was performed using the Wilcoxon rank-sum test.

### Recombinant protein expression and purification

DNA encoding the RING and ROQ domains (RC3H1-N1; aa 2–399) or the RING, ROQ and CCCH-Znf domains (RC3H1-N2; aa 2–452) was subcloned into the pQLinkH vector^54^. The genes were expressed as N-terminal His_7_-tagged proteins at 17 °C in *Escherichia coli* Rosetta 2 (DE3, (Novagen) using a LEX ultra-high-throughput bench-top bioreactor (Harbinger Biotech). Cells were grown at 37 °C in Terrific Broth medium and induced at an OD_600_ of 2.0–2.5 with 0.5 mM isopropyl β-D-1-thiogalactopyranoside. For purification, cells were resuspended in phosphate-buffered saline lysis buffer (1 × phosphate-buffered saline, pH 7.4, 0.5 M NaCl, 5% (v/v) glycerol and 0.5 mM DTT), supplemented with 0.25% (w/v) 3-[(3-cholamidopropyl) dimethylammonio]-1-propanesulfonate, 0.1 mM phenylmethyl sulfonyl fluoride, 1 U ml^−1^ RNase-free DNase I (Qiagen) and one tablet of EDTA-free Complete Protease Inhibitor (Roche). The purification procedure comprises mechanical cell lysis by sonication (SONOPULS HD 2200, Bandelin), an Ni/Zn affinity chromatography on a 5-ml HisTrap FF crude column (GE Healthcare), and a size-exclusion chromatography on a Superdex 200 prep grade column (XK 26 × 60, GE Healthcare). The His_7_ tag was cleaved with tobacco etch virus protease before the gel filtration step, followed by a reapplication of the cleaved protein on the Ni/Zn affinity column. The purification of protein constructs comprising the RING, ROQ and CCCH-Znf domains additionally included a cation-exchange chromatography on a Source 30S column (HR 16 × 10, GE Healthcare).

### Electrophoretic mobility shift assay

The EMSA was performed according to Ryder *et al.*^55^ with the following modifications: RNA was prepared by 5′-end labelling of commercially synthesized RNA oligonucleotides with [γ-^32^P]-ATP using T4 polynucleotide kinase (NEB). Labelled RNA was gel-purified, eluted and adjusted with H_2_O to 1 pmol μl^−1^. Labelled RNA (50 fmol) was used per 20 μl reaction. Before binding reactions, a master mix containing labelled RNA, 1 × binding buffer (20 mM Tris-HCl (pH 7.5), 50 mM KCl, 5 mM MgCl_2_, 20 μM ZnSO_4_ and 10% glycerol), 2 mM DTT, 0.05 mg ml^−1^ BSA and 5 μg ml^−1^ heparin was heated at 90 °C for 1 min and gradually cooled down to room temperature. In parallel, a dilution series of 10 × protein stocks was prepared in 1 × protein dilution buffer (1 × binding buffer and 5 μg ml^−1^ heparin). For each binding reaction, 2 μl of the 10 × protein stock was added to 18 μl of the mastermix at room temperature for 2 h. After addition of 4 μl 6 × loading buffer (30% glycerol, bromophenol blue and xylene cyanol), RNA-protein complexes were resolved by nondenaturing PAGE (6% polyacrylamide, 0.5 × Tris-borate-EDTA (TBE) and 5% glycerol) in ice-cold 0.5 × TBE buffer containing 20 μM ZnSO_4_ at 100 V for 40 min. The protein-bound RNA and the free RNA were quantified using a phosphorimager.

To determine NF-κB DNA-binding activity, EMSA was performed according to Hinz *et al.*^56^ using the following protocol. An amount of 4–10 μg of whole-cell lysate was mixed with radioactive-labelled (25,000 c.p.m.) oligonucleotides containing a NF-κB site (5′-gatcCAGGGCTGGGGATTCCCCATCTCCACAGG-3′ and 5′-gatcCCTGTGGAGATGGGGAATCCCCAGCCCTG-3′), 2 μg poly(dI-dC), 1 μg BSA and 1 mM DTT in 20 μl reaction buffer (20 mM HEPES (pH 8.4), 60 mM KCI and 8% Ficoll) Samples were incubated for 30 min at 25 °C and analysed by native PAGE (5 % gels; TBE buffer), followed by autoradiography.

### Microarray data processing

Microarray raw data for DNA damage response and TNFα response were retrieved from GEO accessions GSE1676 and GSE28548, respectively. Robust multi-array average background correction and quantile normalization was applied using affyR Bioconductor packages^57^. For the analysis of Affymetrix Human Genome U133 Plus 2.0 Array, probe set intensities mapping to the same gene were averaged to summarize into gene intensities, and genes with log2 steady-state expression level <5 were filtered out.

### Mathematical modelling of NF-κB pathway

The computational model of the canonical IKK/NF-κB system is described by an ordinary differential equation system:


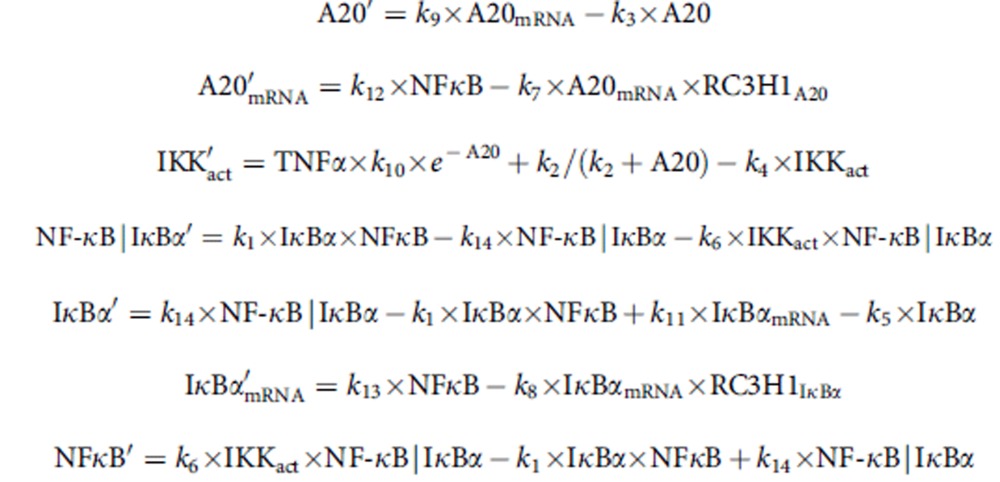


The western blot data of three replicates for phosphorylated IκBα and IKK upon TNFα stimulation with and without RC3H1 induction were quantified using ImageJ. The mRNA levels of IκBα and A20 upon TNFα stimulation with and without RC3H1 induction were measured with qPCR. The parameters of the model were estimated with the Data2Dynamics software package in MATLAB (R2013b, The Mathworks Inc., Natick, MA) using the build-in function *lsqnonlin* and latin hypercube parameter sampling^58^. One representative parameter set is given in Supplementary Table 3.

## Additional information

**Accession Codes:** PAR-CLIP cDNA sequencing and mRNA decay data have been deposited (GEO: GSE69153).

**How to cite this article**: Murakawa, Y. *et al.* RC3H1 post-transcriptionally regulates A20 mRNA and modulates the activity of the IKK/NF-κB pathway. *Nat. Commun.* 6:7367 doi: 10.1038/ncomms8367 (2015).

## Supplementary Material

Supplementary Information Supplementary Figures 1-6, Supplementary Tables 1-3.

Supplementary Dataset 1 RC3H1 target transcripts identified by PAR-CLIP. Table shows weighted number of PAR-CLIP sequence clusters, nucleotide transition events (T to C), fraction of length covered by clusters (percent), and nt covered by PAR-CLIP clusters for each RC3H1 target transcript for the transcript region indicated.

Supplementary Dataset 2 7mer motif analysis. Table shows 7mer sequence, 7mer frequency in 3' UTR PAR-CLIP crosslinked centered regions, frequency of 7mer in background and enrichment for each 7mer.

Supplementary Dataset 3 Global mRNA half-lives measurement. Table shows ENSEMBL Gene Id, Official Gene Symbol, transcript type, half-life (in min) and Minimum Expression Value of transcript of measured mRNA transcripts.

Supplementary Dataset 4 Features of 3'UTR RC3H1 binding sites. Table shows chromosome start/end position and strand, PAR-CLIP ID (only 3' UTR binding sites), sequence of the crosslink centered cluster (CCR), binding cluster, number of diagnostic transition events, gene symbol, rank before normalization based on diagnostic events, mRNA half-live (min), expression level, and expression normalized PAR-CLIP score for each identified PAR-CLIP binding site.

## References

[b1] Schoenberg D.R. & Maquat L.E. Regulation of cytoplasmic mRNA decay. Nat. Rev. Genet. 13, 246–259 (2012). 22392217 10.1038/nrg3160 PMC3351101

[b2] Hao S. & Baltimore D. The stability of mRNA influences the temporal order of the induction of genes encoding inflammatory molecules. Nat. Immunol. 10, 281–288 (2009). 19198593 10.1038/ni.1699 PMC2775040

[b3] Vinuesa C.G. *et al.* A RING-type ubiquitin ligase family member required to repress follicular helper T cells and autoimmunity. Nature 435, 452–458 (2005). 15917799 10.1038/nature03555

[b4] Athanasopoulos V. *et al.* The ROQUIN family of proteins localizes to stress granules via the ROQ domain and binds target mRNAs. FEBS J. 277, 2109–2127 (2010). 20412057 10.1111/j.1742-4658.2010.07628.x

[b5] Pratama A. *et al.* Roquin-2 shares functions with its paralog Roquin-1 in the repression of mRNAs controlling T follicular helper cells and systemic inflammation. Immunity 38, 669–680 (2013). 23583642 10.1016/j.immuni.2013.01.011

[b6] Yu D. *et al.* Roquin represses autoimmunity by limiting inducible T-cell co-stimulator messenger RNA. Nature 450, 299–303 (2007). 18172933 10.1038/nature06253

[b7] Glasmacher E. *et al.* Roquin binds inducible costimulator mRNA and effectors of mRNA decay to induce microRNA-independent post-transcriptional repression. Nat. Immunol. 11, 725–733 (2010). 20639877 10.1038/ni.1902

[b8] Vogel K.U. *et al.* Roquin paralogs 1 and 2 redundantly repress the Icos and Ox40 costimulator mRNAs and control follicular helper T cell differentiation. Immunity 38, 655–668 (2013). 23583643 10.1016/j.immuni.2012.12.004

[b9] Leppek K., Schott J., Reitter S., Poetz F., Hammond M.C. & Stoecklin G. Roquin promotes constitutive mRNA decay via a conserved class of stem-loop recognition motifs. Cell 153, 869–881 (2013). 23663784 10.1016/j.cell.2013.04.016

[b10] Schlundt A. *et al.* Structural basis for RNA recognition in roquin-mediated post-transcriptional gene regulation. Nat. Struct. Mol. Biol. 21, 671–678 (2014). 25026077 10.1038/nsmb.2855

[b11] Tan D., Zhou M., Kiledjian M. & Tong L. The ROQ domain of Roquin recognizes mRNA constitutive-decay element and double-stranded RNA. Nat. Struct. Mol. Biol. 21, 679–685 (2014). 25026078 10.1038/nsmb.2857 PMC4125485

[b12] Schuetz A., Murakawa Y., Rosenbaum E., Landthaler M. & Heinemann U. Roquin binding to target mRNAs involves a winged helix-turn-helix motif. Nat. Commun. 5, 5701 (2014). 25504471 10.1038/ncomms6701

[b13] Maruyama T. *et al.* Roquin-2 promotes ubiquitin-mediated degradation of ASK1 to regulate stress responses. Sci. Signal. 7, ra8 (2014). 24448648 10.1126/scisignal.2004822

[b14] Brooks S.A. & Blackshear P.J. Tristetraprolin (TTP): Interactions with mRNA and proteins, and current thoughts on mechanisms of action. Biochim. Biophys. Acta 1829, 666–679 (2013). 23428348 10.1016/j.bbagrm.2013.02.003 PMC3752887

[b15] Mukherjee N. *et al.* Global target mRNA specification and regulation by the RNA-binding protein ZFP36. Genome Biol. 15, R12 (2014). 24401661 10.1186/gb-2014-15-1-r12 PMC4053807

[b16] Shaw G. & Kamen R. A conserved AU sequence from the 3' untranslated region of GM-CSF mRNA mediates selective mRNA degradation. Cell 46, 659–667 (1986). 3488815 10.1016/0092-8674(86)90341-7

[b17] Caput D., Beutler B., Hartog K., Thayer R., Brown-Shimer S. & Cerami A. Identification of a common nucleotide sequence in the 3'-untranslated region of mRNA molecules specifying inflammatory mediators. Proc. Natl Acad. Sci. USA 83, 1670–1674 (1986). 2419912 10.1073/pnas.83.6.1670 PMC323145

[b18] Chen C.Y. & Shyu A.B. AU-rich elements: characterization and importance in mRNA degradation. Trends Biochem. Sci. 20, 465–470 (1995). 8578590 10.1016/s0968-0004(00)89102-1

[b19] Hafner M. *et al.* Transcriptome-wide identification of RNA-binding protein and microRNA target sites by PAR-CLIP. Cell 141, 129–141 (2010). 20371350 10.1016/j.cell.2010.03.009 PMC2861495

[b20] Shembade N. & Harhaj E.W. Regulation of NF-kappaB signaling by the A20 deubiquitinase. Cell. Mol. Immunol. 9, 123–130 (2012). 22343828 10.1038/cmi.2011.59 PMC3532050

[b21] Ma A. & Malynn B.A. A20: linking a complex regulator of ubiquitylation to immunity and human disease. Nat. Rev. Immunol. 12, 774–785 (2012). 23059429 10.1038/nri3313 PMC3582397

[b22] Lebedeva S. *et al.* Transcriptome-wide analysis of regulatory interactions of the RNA-binding protein HuR. Mol. Cell 43, 340–352 (2011). 21723171 10.1016/j.molcel.2011.06.008

[b23] Ogata H., Goto S., Sato K., Fujibuchi W., Bono H. & Kanehisa M. KEGG: Kyoto Encyclopedia of Genes and Genomes. Nucleic Acids Res. 27, 29–34 (1999). 9847135 10.1093/nar/27.1.29 PMC148090

[b24] Ashburner M. *et al.* Gene ontology: tool for the unification of biology. The Gene Ontology Consortium. Nat. Genet. 25, 25–29 (2000). 10802651 10.1038/75556 PMC3037419

[b25] Heyne S., Costa F., Rose D. & Backofen R. GraphClust: alignment-free structural clustering of local RNA secondary structures. Bioinformatics 28, i224–i232 (2012). 22689765 10.1093/bioinformatics/bts224 PMC3371856

[b26] Will S., Joshi T., Hofacker I.L., Stadler P.F. & Backofen R. LocARNA-P: accurate boundary prediction and improved detection of structural RNAs. RNA 18, 900–914 (2012). 22450757 10.1261/rna.029041.111 PMC3334699

[b27] Will S., Reiche K., Hofacker I.L., Stadler P.F. & Backofen R. Inferring noncoding RNA families and classes by means of genome-scale structure-based clustering. PLoS Comput. Biol. 3, e65 (2007). 17432929 10.1371/journal.pcbi.0030065 PMC1851984

[b28] Yao Z., Weinberg Z. & Ruzzo W.L. CMfinder--a covariance model based RNA motif finding algorithm. Bioinformatics 22, 445–452 (2006). 16357030 10.1093/bioinformatics/btk008

[b29] Nawrocki E.P., Kolbe D.L. & Eddy S.R. Infernal 1.0: inference of RNA alignments. Bioinformatics 25, 1335–1337 (2009). 19307242 10.1093/bioinformatics/btp157 PMC2732312

[b30] Ong S.E., Foster L.J. & Mann M. Mass spectrometric-based approaches in quantitative proteomics. Methods 29, 124–130 (2003). 12606218 10.1016/s1046-2023(02)00303-1

[b31] Doidge R., Mittal S., Aslam A. & Winkler G.S. Deadenylation of cytoplasmic mRNA by the mammalian Ccr4-Not complex. Biochem. Soc. Trans. 40, 896–901 (2012). 22817755 10.1042/BST20120074

[b32] Dolken L. *et al.* High-resolution gene expression profiling for simultaneous kinetic parameter analysis of RNA synthesis and decay. RNA 14, 1959–1972 (2008). 18658122 10.1261/rna.1136108 PMC2525961

[b33] Selbach M., Schwanhausser B., Thierfelder N., Fang Z., Khanin R. & Rajewsky N. Widespread changes in protein synthesis induced by microRNAs. Nature 455, 58–63 (2008). 18668040 10.1038/nature07228

[b34] Elkon R., Linhart C., Sharan R., Shamir R. & Shiloh Y. Genome-wide in silico identification of transcriptional regulators controlling the cell cycle in human cells. Genome Res. 13, 773–780 (2003). 12727897 10.1101/gr.947203 PMC430898

[b35] Napetschnig J. & Wu H. Molecular basis of NF-kappaB signaling. Ann. Rev. Biophys. 42, 443–468 (2013). 23495970 10.1146/annurev-biophys-083012-130338 PMC3678348

[b36] Huang T.T., Wuerzberger-Davis S.M., Wu Z.H. & Miyamoto S. Sequential modification of NEMO/IKKgamma by SUMO-1 and ubiquitin mediates NF-kappaB activation by genotoxic stress. Cell 115, 565–576 (2003). 14651848 10.1016/s0092-8674(03)00895-x

[b37] Grimley R. *et al.* Over expression of wild type or a catalytically dead mutant of Sirtuin 6 does not influence NFkappaB responses. PLoS ONE 7, e39847 (2012). 22792191 10.1371/journal.pone.0039847 PMC3391194

[b38] Renner F. & Schmitz M.L. Autoregulatory feedback loops terminating the NF-kappaB response. Trends Biochem. Sci. 34, 128–135 (2009). 19233657 10.1016/j.tibs.2008.12.003

[b39] Hayden M.S. & Ghosh S. NF-kappaB, the first quarter-century: remarkable progress and outstanding questions. Genes Dev. 26, 203–234 (2012). 22302935 10.1101/gad.183434.111 PMC3278889

[b40] Hinz M. & Scheidereit C. The IkappaB kinase complex in NF-kappaB regulation and beyond. EMBO Rep. 15, 46–61 (2014). 24375677 10.1002/embr.201337983 PMC4303448

[b41] Ray D. *et al.* A compendium of RNA-binding motifs for decoding gene regulation. Nature 499, 172–177 (2013). 23846655 10.1038/nature12311 PMC3929597

[b42] Bertossi A. *et al.* Loss of Roquin induces early death and immune deregulation but not autoimmunity. J. Exp. Med. 208, 1749–1756 (2011). 21844204 10.1084/jem.20110578 PMC3171092

[b43] Walle L.V. *et al.* Negative regulation of the NLRP3 inflammasome by A20 protects against arthritis. Nature 512, 69–73 (2014). 25043000 10.1038/nature13322 PMC4126806

[b44] Baltz A.G. *et al.* The mRNA-bound proteome and its global occupancy profile on protein-coding transcripts. Mol. Cell 46, 674–690 (2012). 22681889 10.1016/j.molcel.2012.05.021

[b45] Schwanhausser B., Gossen M., Dittmar G. & Selbach M. Global analysis of cellular protein translation by pulsed SILAC. Proteomics 9, 205–209 (2009). 19053139 10.1002/pmic.200800275

[b46] Huang da W., Sherman B.T. & Lempicki R.A. Systematic and integrative analysis of large gene lists using DAVID bioinformatics resources. Nat. Protoc. 4, 44–57 (2009). 19131956 10.1038/nprot.2008.211

[b47] Huang da W., Sherman B.T. & Lempicki R.A. Bioinformatics enrichment tools: paths toward the comprehensive functional analysis of large gene lists. Nucleic Acids Res. 37, 1–13 (2009). 19033363 10.1093/nar/gkn923 PMC2615629

[b48] Grillo G. *et al.* UTRdb and UTRsite (RELEASE 2010): a collection of sequences and regulatory motifs of the untranslated regions of eukaryotic mRNAs. Nucleic Acids Res. 38, D75–D80 (2010). 19880380 10.1093/nar/gkp902 PMC2808995

[b49] Hofacker I.L. RNA secondary structure analysis using the Vienna RNA package. Curr. Protoc. Bioinformatics Chapter 12, Unit 12 2 (2004). 18428716 10.1002/0471250953.bi1202s04

[b50] Rappsilber J., Ishihama Y. & Mann M. Stop and go extraction tips for matrix-assisted laser desorption/ionization, nanoelectrospray, and LC/MS sample pretreatment in proteomics. Anal. Chem. 75, 663–670 (2003). 12585499 10.1021/ac026117i

[b51] Cox J., Neuhauser N., Michalski A., Scheltema R.A., Olsen J.V. & Mann M. Andromeda: a peptide search engine integrated into the MaxQuant environment. J. Proteome Res. 10, 1794–1805 (2011). 21254760 10.1021/pr101065j

[b52] Kim D., Pertea G., Trapnell C., Pimentel H., Kelley R. & Salzberg S.L. TopHat2: accurate alignment of transcriptomes in the presence of insertions, deletions and gene fusions. Genome Biol. 14, R36 (2013). 23618408 10.1186/gb-2013-14-4-r36 PMC4053844

[b53] Gaidatzis D., Lerch A., Hahne F. & Stadler M.B. QuasR: quantification and annotation of short reads in R. Bioinformatics 31, 1130–1132 (2014). 25417205 10.1093/bioinformatics/btu781 PMC4382904

[b54] Scheich C., Kummel D., Soumailakakis D., Heinemann U. & Bussow K. Vectors for co-expression of an unrestricted number of proteins. Nucleic Acids Res. 35, e43 (2007). 17311810 10.1093/nar/gkm067 PMC1874614

[b55] Ryder S.P., Recht M.I. & Williamson J.R. Quantitative analysis of protein-RNA interactions by gel mobility shift. Methods Mol. Biol. 488, 99–115 (2008). 18982286 10.1007/978-1-60327-475-3_7 PMC2928675

[b56] Hinz M., Stilmann M., Arslan S.C., Khanna K.K., Dittmar G. & Scheidereit C. A cytoplasmic ATM-TRAF6-cIAP1 module links nuclear DNA damage signaling to ubiquitin-mediated NF-kappaB activation. Mol. Cell 40, 63–74 (2010). 20932475 10.1016/j.molcel.2010.09.008

[b57] Gautier L., Cope L., Bolstad B.M. & Irizarry R.A. affy--analysis of Affymetrix GeneChip data at the probe level. Bioinformatics 20, 307–315 (2004). 14960456 10.1093/bioinformatics/btg405

[b58] Raue A. *et al.* Lessons learned from quantitative dynamical modeling in systems biology. PLoS ONE 8, e74335 (2013). 24098642 10.1371/journal.pone.0074335 PMC3787051

