# Supplementary material for: RC3H1 post-transcriptionally regulates A20 mRNA and modulates the activity of the IKK/NF-κB pathway
Source: Nat Commun. 2015 Jul 14;6:7367. doi: 10.1038/ncomms8367 (PMC4510711; doi:10.1038/ncomms8367)
Supplement: Supplementary Information — Supplementary Figures 1-6, Supplementary Tables 1-3. [file ncomms8367-s1.pdf]

SUPPLEMENTARY FIGURES

Supplementary Figure-1 (Landthaler et al.)

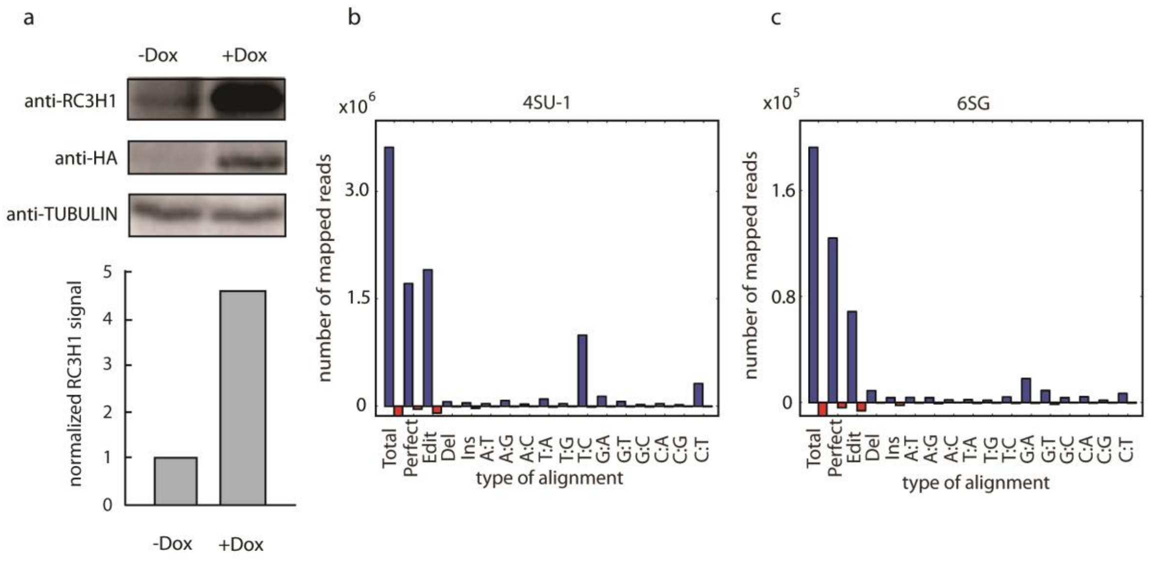

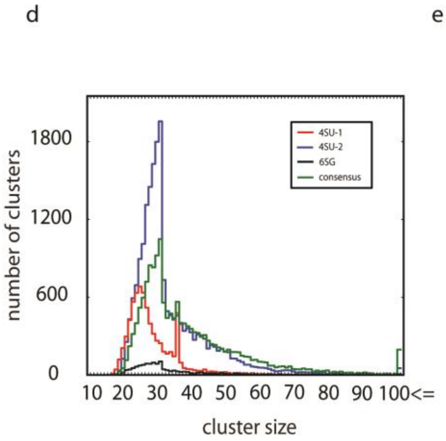

e

KEGG pathway	count	Benjamini
Chronic myeloid leukemia	16	5.3E-4
Cell cycle	19	3.5E-3
Colorectal cancer	13	4.5E-2
TGF-beta signaling pathway	13	4.6E-2
Prostate cancer	13	3.8E-2
Ubiquitin mediated proteolysis	17	3.9E-2
p53 signaling pathway	11	4.0E-2
Pathways in cancer	30	3.7E-2
Melanoma	10	1.3E-1
Endocytosis	18	1.5E-1

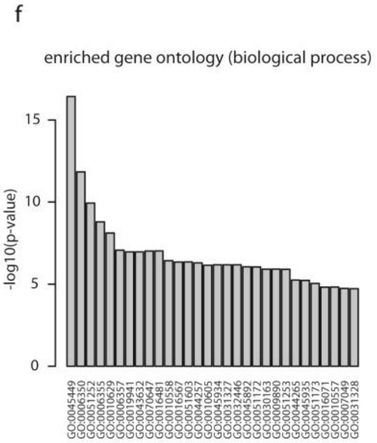

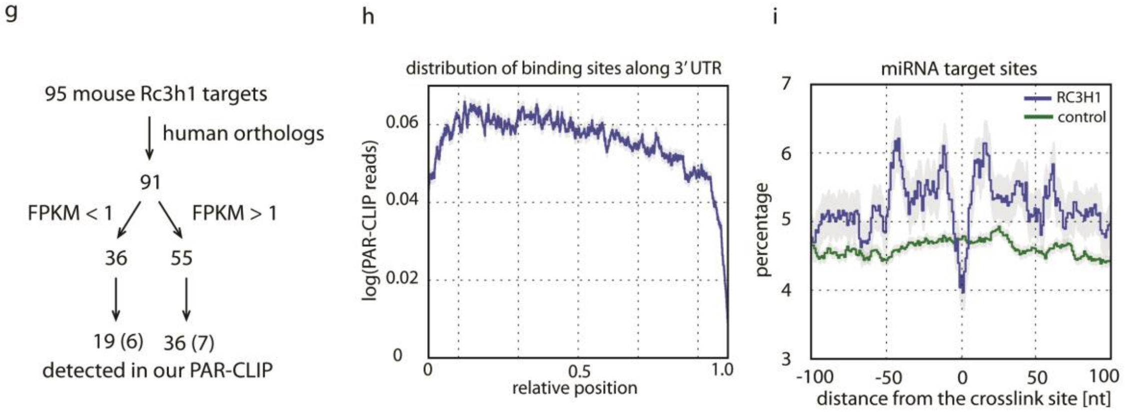

Supplementary Figure 1

(a) Western blot analyses for RC3H1, FLAG/HA-RC3H1, and TUBULIN in the presence and absence of doxycycline (1 μg/ml for 9 hours). RC3H1 signal was quantified and normalized for TUBULIN loading control. A bar plot shows relative normalized expression level of RC3H1.

(b) The frequency of nucleotide mismatches in 4SU-1 PAR-CLIP reads aligned to mature mRNAs by PAR-CLIP analysis pipeline is shown. Sense mapping is shown in blue and antisense mapping in red.

(c) The frequency of nucleotide mismatches in 6SG PAR-CLIP reads aligned to mature mRNAs by PAR-CLIP analysis pipeline is shown. Sense mapping is shown in blue and antisense mapping in red.

(d) A length histogram of clusters identified in the following PAR-CLIP experiments: 4SU-1 (red), 4SU-2 (blue), 6SG (black) or consensus (green). RC3H1 PAR-CLIP clusters are short with a median cluster length of around 25 to 30 nt.

(e) Top 1000 "consensus set" target genes, ranked by the number of transitions in the 3'UTR, were subjected to enrichment analysis for the KEGG pathway using the on-line DAVID program. The top 10 KEGG pathways are shown. The number of genes falling into each pathway and p-values corrected for multiple comparisons according to Benjamini-Hochberg are shown in "count" and "Benjamini" column, respectively.

(f) Top 1000 "consensus set" target genes, ranked by the number of transitions in the 3'UTR, were subjected to the enrichment analysis for the GO term (Biological Processes) using the on-line DAVID program. The top 30 biological processes are shown. Y axis shows –log10 of p-values corrected for multiple comparison by the Benjamini-Hochberg method. Following are the details of GO terms: GO:0045449; regulation of transcription, GO:0006350; transcription, GO:0051252; regulation of RNA metabolic process, GO:0006355; regulation of transcription, DNA-dependent, GO:0010629; negative regulation of gene expression, GO:0006357; regulation of transcription from RNA polymerase II promoter, GO:0019941; modification-dependent protein catabolic process, GO:0043632; modification-dependent macromolecule catabolic process, GO:0070647; protein modification by small protein conjugation or removal, GO:0016481; negative regulation of transcription, GO:0010558; negative regulation of macromolecule biosynthetic process, GO:0016567; protein ubiquitination, GO:0051603; proteolysis involved in cellular protein catabolic process, GO:0044257; cellular protein catabolic process, GO:0010605; negative regulation of macromolecule metabolic process, GO:0045934; negative regulation of nucleobase, nucleoside, nucleotide and nucleic acid metabolic process, GO:0031327; negative regulation of cellular biosynthetic process, GO:0032446; protein modification by small protein conjugation, GO:0045892; negative regulation of transcription, DNA-dependent, GO:0051172; negative regulation of nitrogen compound metabolic process, GO:0030163; protein catabolic process, GO:0009890; negative regulation of biosynthetic process, GO:0051253; negative regulation of RNA metabolic process, GO:0044265; cellular macromolecule catabolic process, GO:0045935; positive regulation of nucleobase, nucleoside, nucleotide and nucleic acid metabolic process, GO:0051173; positive regulation of nitrogen compound metabolic process,

GO:0016071; mRNA metabolic process, GO:0010557; positive regulation of macromolecule biosynthetic process, GO:0007049; cell cycle, GO:0031328; positive regulation of cellular biosynthetic process.

(g) Mouse RC3H1 target mRNAs identified by Leppek and colleagues are compared to human PAR-CLIP RC3H1 target mRNAs. Out of 95 genes, 91 genes are converted to orthologous human genes, and divided into two groups based on FPKM expression value in HEK293 cells. For each group, number of mRNAs that are overlapping in human PAR-CLIP RC3H1 target mRNAs is shown. Number of mouse CDE containing mRNAs is shown in parentheses.

(h) Distribution of consensus RC3H1 binding cluster along 3’UTRs of mRNA.

(i) Density of predicted conserved miRNA target sites around crosslink sites in 3’UTRs. RC3H1 crosslink sites and miRNA target sites display no tendency for direct overlap but the larger context (10–50 nt) shows mildly elevated seed density. The gray envelope represents the standard error of the mean. RC3H1 target sites identified by the 4SU-1 PAR-CLIP were used in this analysis.

Supplementary Figure-2 (Landthaler et al.)

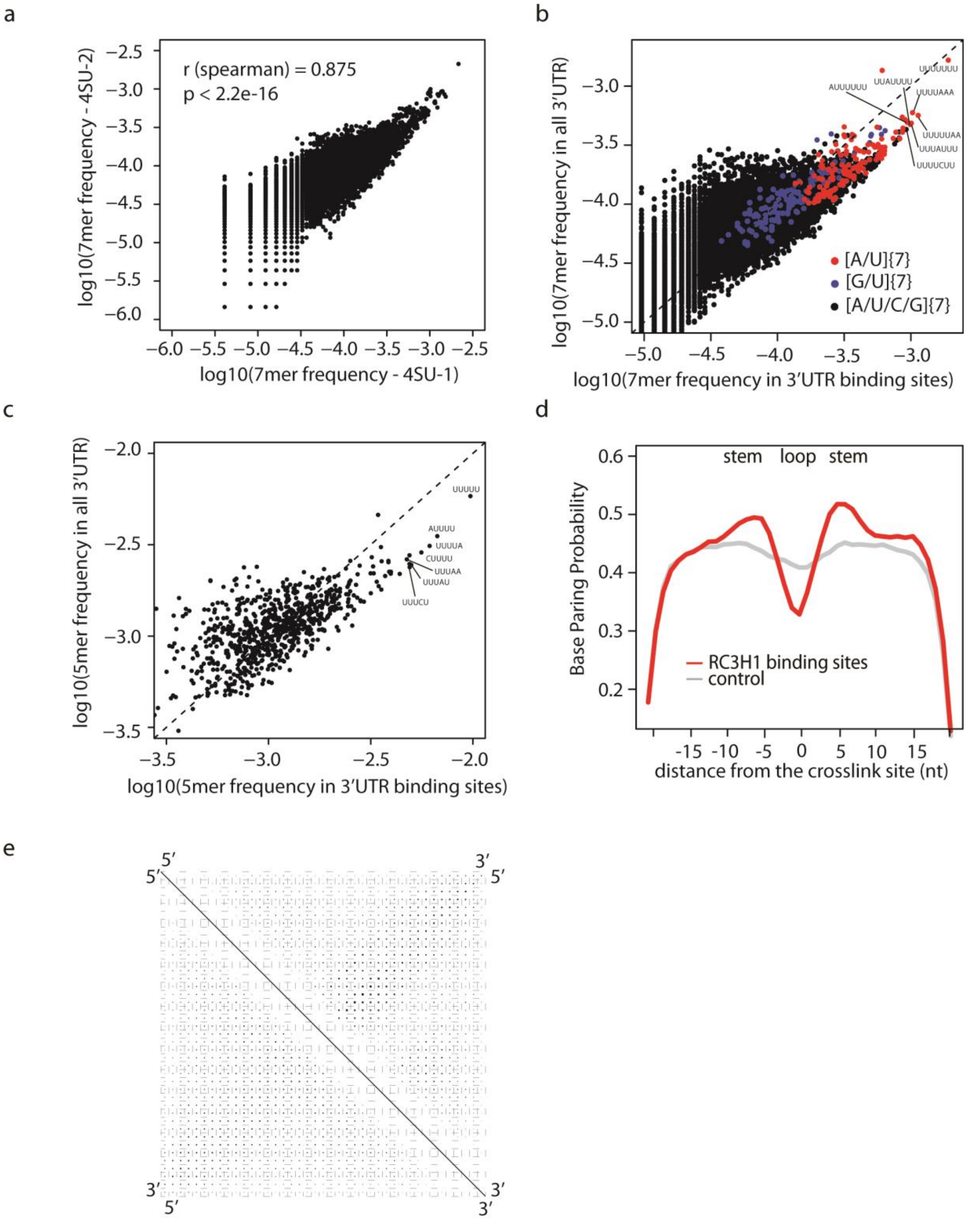

Supplementary Figure 2

(a) log10 frequencies of 7mers occurring in the 41 nt window around the RC3H1 preferred crosslink sites are shown for 4SU-1 PAR-CLIP and 4SU-2 PAR-CLIP libraries, showing a good correlation of 7mer occurrence between the libraries.

(b) A scatter plot showing 7mers log10 frequencies in the 41 nt window around the preferred crosslink sites of consensus 3'UTR RC3H1 binding sites versus 7mers log10 frequencies in all 3'UTR sequences. 7mers comprising of only A/U or G/U are plotted in red or blue, respectively. U-rich sequences with A contents (red) are more frequent and enriched over the background frequency compared to control 7mer U-rich sequences with G contents.

(c) A scatter plot showing 5mers log10 frequencies in the 41 nt window around the preferred crosslink sites of consensus 3'UTR RC3H1 binding sites versus 5mers log10 frequencies in all 3'UTR sequences.

(d) RC3H1 binding sites tend to have stem-loop secondary structure. 41 nt sequences centered around RC3H1 crosslink site were computationally folded. Base pairing probability for each position around crosslink sites are averaged over all 3'UTR binding site (red) and control 3'UTR sequences (gray).

(e) A RNA structure dot plot for top1000 RC3H1 consensus targets, ranked by number of T to C transition events in 3'UTR (top right triangular), and control sets of random 3'UTR sequence from RC3H1 target genes (bottom left triangular) demonstrates the stem-loop structure of RC3H1 binding sites. A dot placed in the *i*th row and *j*th column of a triangular array represents the base pair between the *i*th base with *j*th base, and the size of dot is proportional to the square root of average base paring probability for each base paring.

Supplementary Figure-3 (Landthaler et al.)

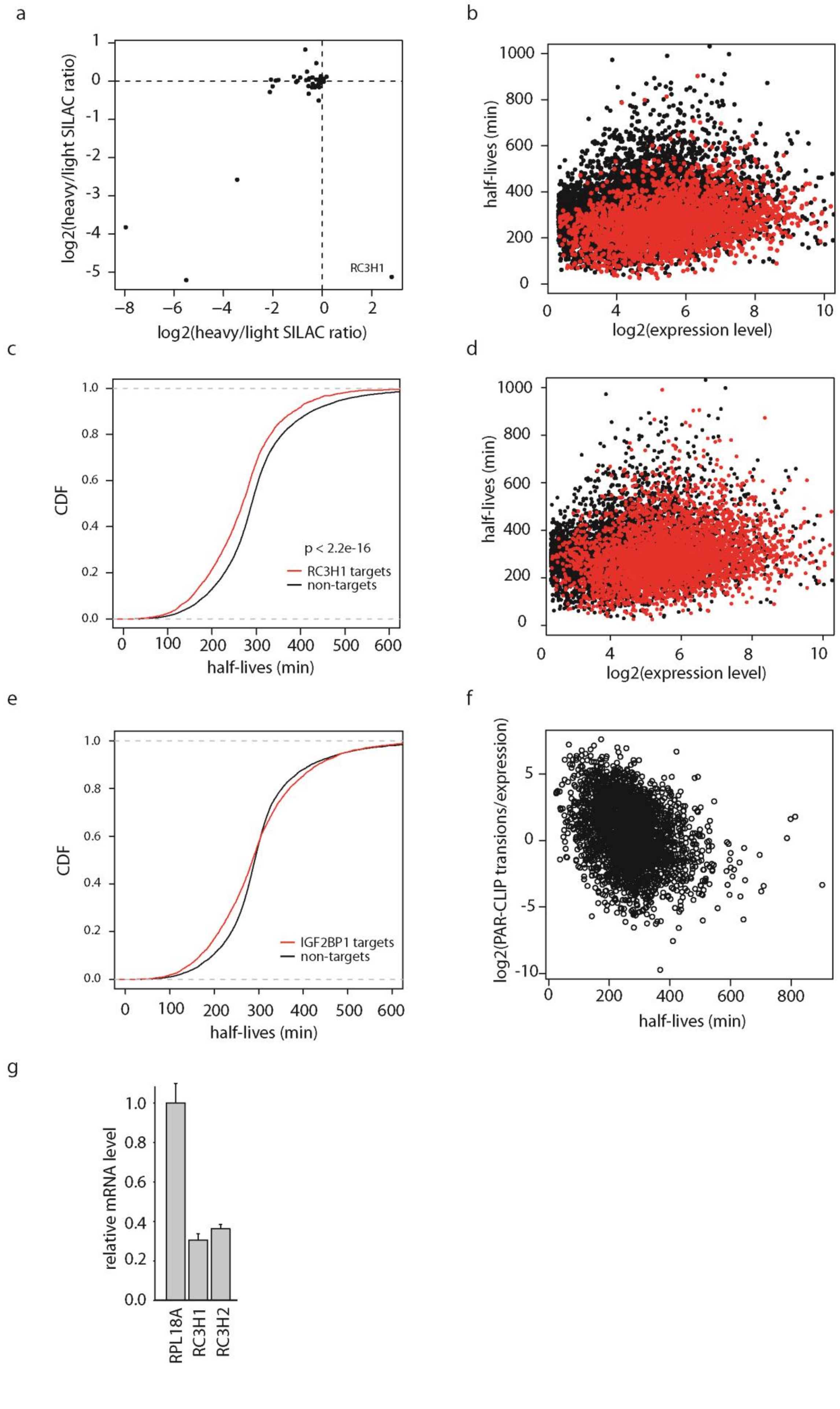

Supplementary Figure 3

(a) A scatter plot of the log2 fold changes of "heavy" to "light" SILAC ratios (H/L) versus "heavy" to "light" SILAC ratios (H/L) in label swap experiment in Figure 3a.

(b) RC3H1 target transcripts have shorter half-lives. Red or black dots represent the 3'UTR RC3H1 consensus targets or non-targets, respectively. log2 expression levels and half-lives (min) are blotted on x- and y-axis, respectively.

(c) A cumulative distribution function (CDF) plot of mRNA half-lives shown in (b). The mean mRNA half-lives of RC3H1 targets and non-targets are 269.9 min and 311.1 min, respectively. The difference is significant with a p-value smaller than 2.2e-16 (Wilcoxon's rank sum test).

(d) IGF2BP1 target transcripts are not as short half-lived as RC3H1 bound mRNAs. Red or black dots represent the 3'UTR IGF2BP1 targets or non-targets, respectively. Log2 expression levels and half-lives (min) are blotted on x- and y-axis, respectively.

(e) CDF plot of mRNA half-lives shown in (d). The mean mRNA half-lives of IGF2BP1 targets and non-targets are 296.3 min and 303.1 min, respectively.

(f) Inverse correlation (Spearman r coefficient -0.33, p value < 2.2e-16) between mRNA half-lives and the PAR-CLIP index, defined by number of RC3H1 PAR-CLIP transitions normalized by the expression levels for each gene.

(g) Quantitative RT-PCR analysis showing that RC3H1 and RC3H2 mRNAs are significantly reduced 3 days after the treatment with siRNAs against RC3H1 (siRNA2) and RC3H2. Relative mRNA expression levels are shown. Average and standard deviation (error bar) from three technical replicates are shown.

Supplementary Figure-4 (Landthaler et al.)

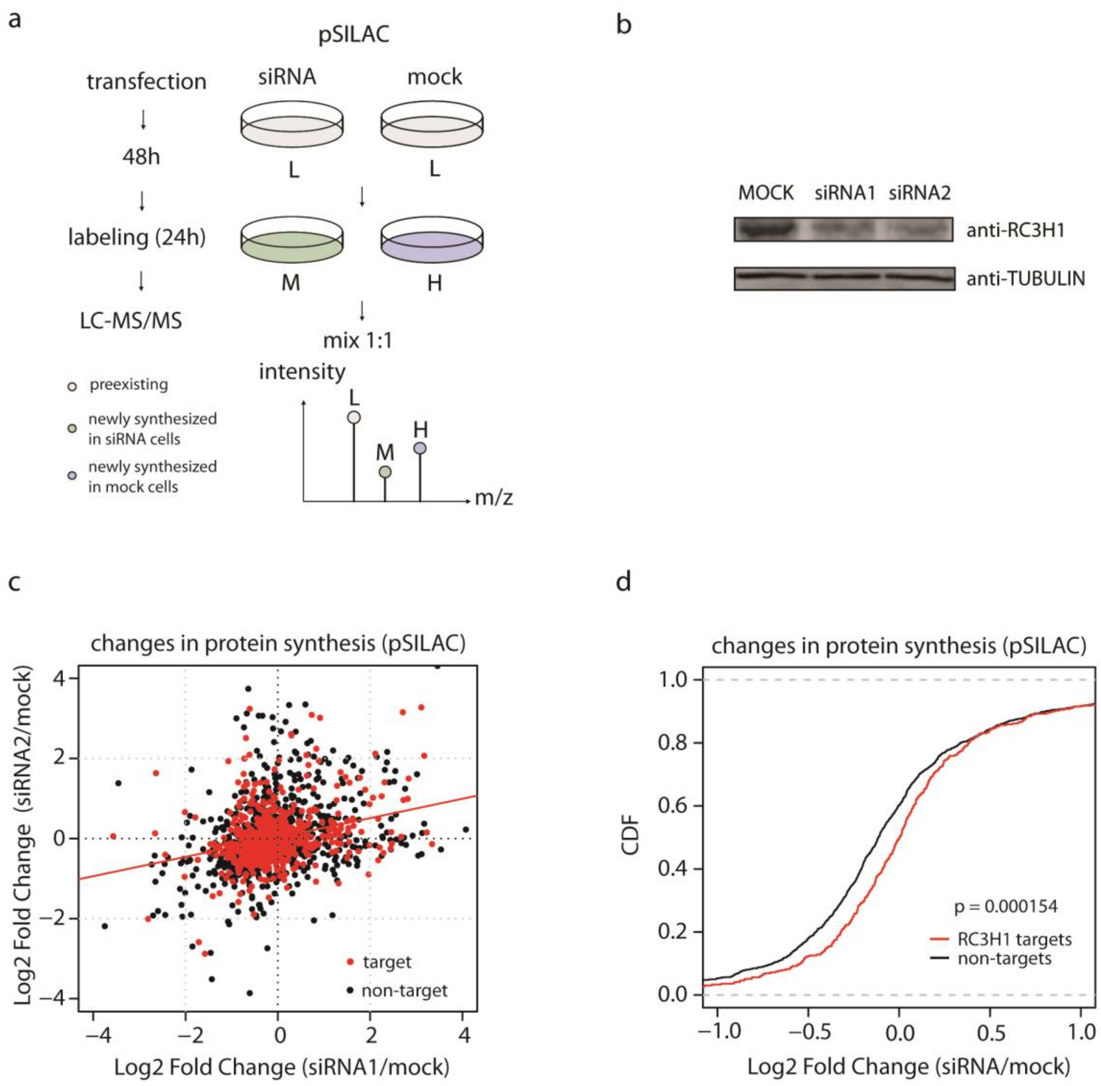

Supplementary Figure 4

(a) Overview of the pulsedSILAC experiment that measures changes in protein synthesis. Cellular proteins incorporate either heavy (mock) or medium-heavy (RC3H1 knockdown) amino acids for 24 h. The mass shift allows measurement of the difference in newly synthesized protein between normal and RC3H1 depleted cells.

(b) Western Blot analyses for endogenous RC3H1 knock-down mediated by two distinct siRNAs (siRNA-1 and siRNA-2) against RC3H1 and for TUBULIN as a loading control.

(c) A scatter plot of log2 fold changes of protein synthesis after siRNA-1 mediated knockdown versus siRNA-2 mediated knockdown. Red and black dots indicate the PAR-CLIP targets (consensus set) and non-targets, respectively (upper right).

(d) A CDF plot of log2 fold changes of protein synthesis of consensus RC3H1 targets that have more than 100 transitions detected in their 3'UTRs (1561 genes) shown in red and non-targets shown in black after siRNA-2 mediated knockdown. Protein synthesis of RC3H1 targets is mildly but statistically significantly increased upon RC3H1 knockdown (p-value 0.00015, Wilcoxon's rank sum test). The mean log2 fold changes of RC3H1 targets (n = 390) and non-targets (n = 1307) are 0.089 and -0.023, respectively.

Supplementary Figure-5 (Landthaler et al.)

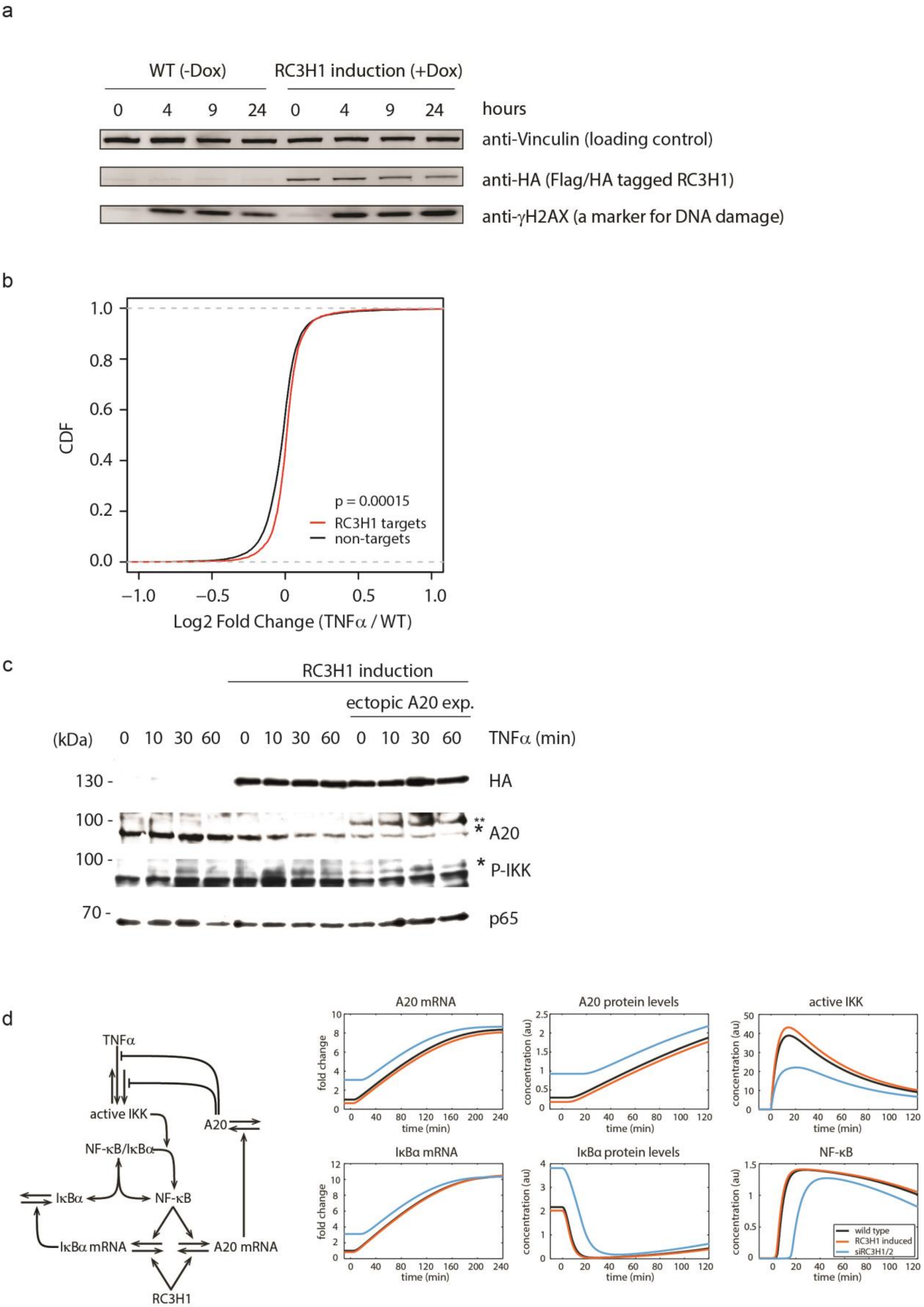

Supplementary Figure 5

(a) Western blot analyses for FLAG/HA-RC3H1, γH2AX (a marker for DNA damage), and vinculin (loading control). Samples are harvested at indicated time points following NCS induced DNA damage. Increased γH2AX indicates the proper induction of DNA damage.

(b) A CDF plot of log2 fold changes upon TNFα treatment (Figure 6a) is shown for RC3H1 3'UTR targets in red and for non-targets in black. The mean log2 fold changes of RC3H1 targets (n = 3184) and non-targets (n = 10142) are 0.0160 and -0.0146, respectively. The difference is significant with p-value of 0.00015 (Wilcoxon's rank sum test).

(c) Western blot analyses of the NF-κB pathway proteins after TNFα stimulation in cells with doxycycline-dependent RC3H1 expression in the presence or the absence of A20 overexpression. HEK293 cells were treated with doxycycline (1 µg/ml; overall for 72 h). After 48h cells were transfected with plasmids expressing a FLAG-tagged and 3'UTR deficient A20 mutant. 24 h. later, cells were treated with TNFα for the indicated times, and analyzed by Western blotting. Increased IKK activation (T-loop phosphorylation, P-IKK) upon ectopic expression of RC3H1 was suppressed by overexpressing A20. For A20, * indicates endogenous A20 and ** indicates FLAG-tagged A20. For P-IKK, * indicates phosphorylated form of IKK.

(c) Western blot analyses of the NF-κB pathway proteins after TNFα stimulation in cells with DOX-dependent RC3H1 expression in the presence or the absence of A20 overexpression. HEK293 cells were treated with doxycycline (1 µg/ml; overall for 72 h). After 48h cells were transfected with plasmids expressing a FLAG-tagged A20 without 3'UTR. 24 h. later, cells were treated with TNFα for the indicated times, and analyzed by Western blotting. Increased IKK activation (T-loop phosphorylation, P-IKK) upon ectopic expression of RC3H1 was suppressed by overexpressing A20. For A20, * indicates endogenous A20 and ** indicates FLAG-tagged A20. For P-IKK, * indicates phosphorylated form of IKK.

(d) Left: Scheme of the mathematical model of the canonical NF-κB pathway and the effect of RC3H1. IKK is basally activated as well as transiently activated by TNFα stimulation. Both processes are inhibited by A20 protein. NF-κB release from the NF-κB/IκBα complex occurs with a basal rate and is induced by activated IKK. Free NF-κB activates IκBα and A20 mRNA transcription. Synthesized IκBα protein binds to NF-κB to form the NF-κB/IκBα complex and thus inactivates NF-κB. Synthesized A20 protein inhibits IKK activity. RC3H1 destabilizes the IκBα and A20 mRNAs. Right: The simulated dynamics of 6 components of the NF-κB model are shown for wild type cells (black lines), RC3H1 overexpression (orange lines) and siRC3H1/2 expression (blue lines). The simulations qualitatively fit to the experimental findings for the three conditions (compare Fig. 6c-f). RC3H1 overexpression (orange lines) leads to a decrease in A20 expression and increases IKK activation (top panels), while marginally effecting IκBα expression and NF-κB activity (bottom panels) compared to wild type cells (black lines). In contrast, siRC3H1/2 treated cells (blue lines) exhibit higher levels of A20 and IκBα and decreased IKK as well as NF-κB activity compared to wild

type cells (black lines).

Supplemeantary Figure-6 (Landthaler et al.)

Figure 1a

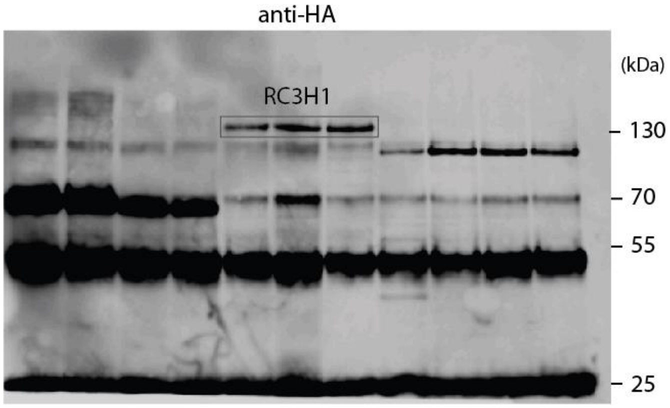

Figure 3b

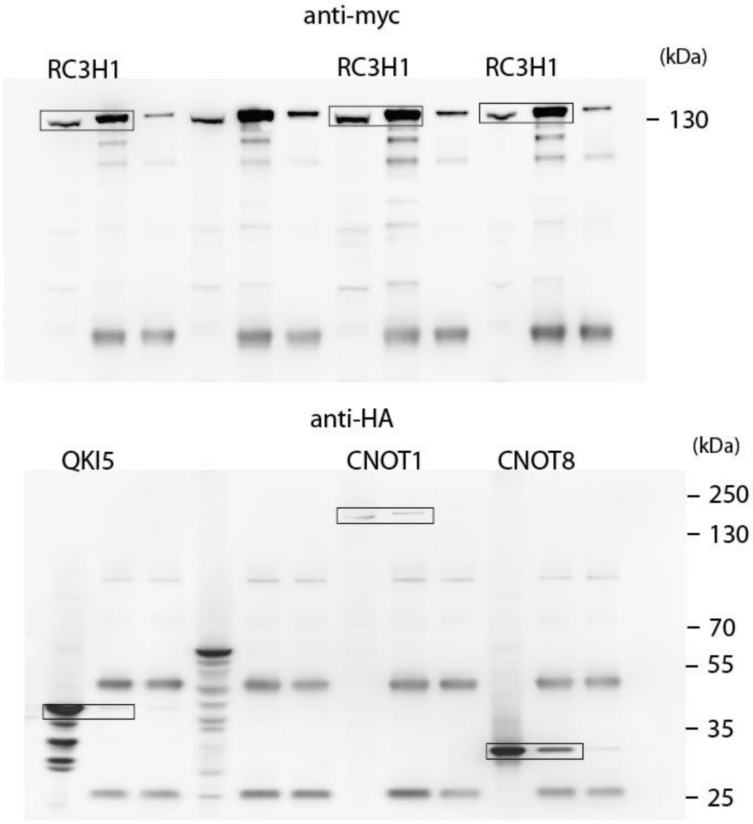

Figure 6c

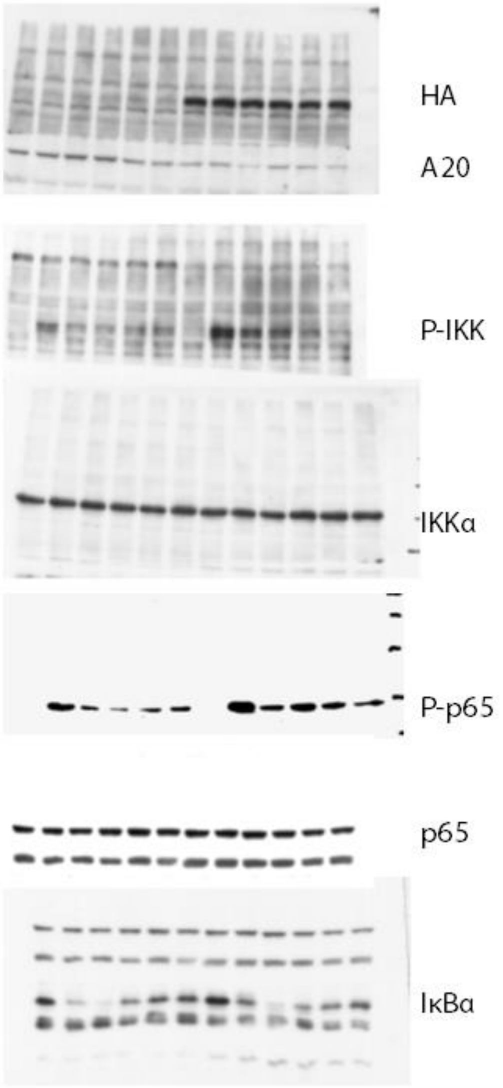

Figure 6e

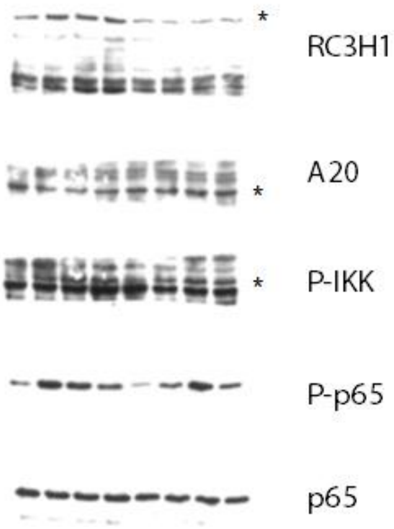

Supplementary Figure 6

Uncropped images of Western blots shown in the Figures 1a, 3b, 6c, and 6e are shown. Antibodies used for Western analyses are indicated on top for Figures 1a and 3b and position of the size markers on the right.

Antibodies used for Figures 6c and 6e are indicated on the right.

SUPPLEMENTARY TABLES

Supplementary Table 1

PAR-CLIP reads mapping statistics

4SU-1 library	all reads	unique reads
Reads mapped	6529788	432984
UCSC genes (exons)	2736088	187329
tRNA	117947	4668
rRNA	1153757	40634
TC-Reads mapped	2352313	114661
TC-Reads UCSC exons	1685256	34259
TC-Reads tRNA	7400	1648
TC-Reads rRNA	106547	5963

4SU-2 library	all reads	unique reads
Reads mapped	1747196	315238
UCSC genes (exons)	1253022	221508
tRNA	12672	1400
rRNA	21087	3041
TC-Reads mapped	1143108	187959
TC-Reads UCSC exons	891261	146441
TC-Reads tRNA	4232	404
TC-Reads rRNA	6084	590

6SG library	all reads	unique reads
Reads mapped	806102	116912
UCSC genes (exons)	402030	67581
tRNA	14264	1762
rRNA	43011	6831
GA-Reads mapped	59902	12588
GA-Reads UCSC exons	34259	8859
GA-Reads tRNA	1648	213
GA-Reads rRNA	2145	456

Reads from individual PAR-CLIP experiment were independently mapped to the human genome (hg18) using tophat2. Mapped all or unique reads in exons of UCSC genes as well as tRNA and rRNA regions were counted using quasR. tRNA and rRNA regions were obtained from the RepeatMasker track of the UCSC genome browser. Reads containing T-to-C transitions for 4SU library (indicated as TC-reads) or G-to-A transitions for 6SG library (indicated as GA reads) were counted and shown in the table.

Supplementary Table 2

RC3H1 interactors identified by SILAC proteomics

GeneSymbol	Peptides count (RC3H1:H, Ctr:L)	Peptides count (RC3H1:L, Ctr:H)	normalized ratio (H/L) (RC3H1:H, Ctr:L)	normalized ratio (H/L) (RC3H1:L, Ctr:H)
RC3H1	68	85	6.9745	0.028621
CNOT1	21	30	0.75969	1.0728
CLASP2	13	17	0.67727	0.91487
STK38	13	13	0.8448	1.3884
CNOT2	12	16	0.23727	1.0303
CLASP2	10	12	0.49829	1.0084
CNOT3	10	15	0.63473	1.0179
KIAA1543	10	15	0.229	0.82195
PRPF31	9	9	0.83936	1.0312
HSPA5	8	11	0.89498	0.94728
SNRPD3	8	8	1.0434	1.045
STK38L	8	6	0.62142	1.7758
SRRM2	7	11	0.24763	0.91045
RPS3	6	8	1.0185	1.0232
ACTG1	5	6	0.83416	0.91681
EIF4B	5	10	0.99178	1.0466
RPS11	5	5	0.76615	0.95361
RPS20	5	7	0.91823	0.94982
TRIM21	5	8	1.0145	1.0401
CNOT8	4	6	0.69573	1.0562
RPS16	4	4	1.0435	0.99228
RPS19	4	6	1.1229	1.0854
RPS6	4	5	0.65036	1.1877
ALB	3	4	0.091675	0.16721
CNOT7	3	4	0.89102	1.032
HNRNPU	3	7	0.47959	0.98462
KRT2	3	2	NA	NA
RC3H2	3	7	NA	NA
RPS17	3	4	0.93651	0.99386
RPS2	3	3	0.92047	0.89879
RQCD1	3	4	0.92335	0.98199
SFPQ	3	5	0.29161	1.0231
ATP5A1	2	2	1.0495	0.90807
C2orf29	2	3	0.93005	0.89471
EDF1	2	2	0.41973	0.85228
FAU	2	2	0.74622	1.0801
GCN1L1	2	3	NA	1.2164
HIST1H1C	2	3	NA	NA

HSPA6	2	2	NA	NA
KRT9	2	0	NA	NA
LARP1	2	4	0.36284	0.92239
MCM7	2	5	0.51008	0.88918
MGA	2	3	0.53502	0.98609
NCL	2	3	1.0548	1.061
NONO	2	3	NA	1.1285
OTUD4	2	3	0.48627	0.85355
PRDX4	2	2	0.72622	0.89963
RAN	2	2	1.0471	1.055
RPL11	2	2	1.0298	0.93367
RPL12	2	2	0.85682	0.66846
RPL22	2	3	0.53273	1.0189
RPL23A	2	4	0.87767	1.0195
RPL27A	2	2	0.53698	1.0883
RPL28	2	3	0.4996	1.0331
RPL38	2	2	0.8659	1.0732
RPS10	2	3	1.3193	1.0228
RPS14	2	3	1.0792	0.83625
TNKS1BP1	2	10	0.61396	1.0045
VARS	2	2	0.24794	0.9639
VIM	2	3	0.51781	0.86893
C11orf84	1	2	NA	0.90834
CAD	1	3	NA	0.97571
CCT6A	1	0	NA	NA
CD2BP2	1	1	NA	NA
CIRBP	1	5	NA	1.2274
CKM	1	0	NA	NA
CMAS	1	1	0.83305	0.74961
CNOT6L	1	0	NA	NA
DDX46	1	1	NA	NA
E2F7	1	1	NA	2.0934
EIF4G1	1	1	NA	1.0222
FARSA	1	2	NA	1.1113
HNRNPA2B1	1	0	NA	NA
HNRNPK	1	2	NA	1.2671
HSPD1	1	2	NA	0.72484
IRS4	1	2	0.37125	0.95832
KIAA1967	1	1	NA	1.0193
KRT5	1	0	NA	NA
LUC7L2	1	1	NA	0.99149
MAP7	1	2	NA	1.0228
NCAPH	1	2	NA	0.78994
NPM1	1	3	1.2018	1.1622
NUFIP2	1	1	NA	NA
OGT	1	1	NA	NA

PABPN1	1	0	NA	NA
PEG10	1	1	NA	NA
PHGDH	1	1	NA	NA
PPIA	1	0	NA	NA
PPIB	1	0	0.23889	NA
PRPF6	1	1	NA	NA
PRSS3	1	1	NA	0.020393
PSMC3	1	1	NA	NA
PSMD11	1	1	NA	1.0896
PUM1	1	1	NA	0.73838
RAVER1	1	2	NA	0.83177
RBM3	1	1	0.25125	0.95073
RCC2	1	1	NA	NA
RNF219	1	6	NA	1.3495
RPL13	1	5	NA	1.0434
RPL24	1	2	NA	1.1117
RPL30	1	2	NA	1.1479
RPS13	1	1	NA	NA
RPS15A	1	1	NA	NA
RPS24	1	1	NA	NA
RPS3A	1	2	NA	1.179
RPS5	1	2	NA	0.99753
RPS7	1	3	NA	0.96961
RPS8	1	4	0.21898	1.0226
SEC16A	1	1	NA	NA
SF3B2	1	2	NA	1.0072
SLC25A3	1	1	NA	1.0013
SLC25A5	1	1	NA	NA
SMC2	1	1	NA	0.99501
SNRNP200	1	1	NA	NA
SNRPD2	1	1	NA	NA
TRIM28	1	3	NA	0.85107
TYRP1	1	0	1.4161	NA
WIZ	1	1	NA	0.86603
ZFP161	1	1	NA	NA
ZNF326	1	1	NA	NA
ACTBL2	0	2	NA	0.62679
AKAP8L	0	1	NA	NA
ATP5C1	0	1	NA	NA
CCDC124	0	2	NA	1.0067
CNOT10	0	1	NA	0.88371
CSDA	0	2	NA	NA
DCD	0	1	NA	NA
DDX17	0	1	NA	NA
EEF1D	0	1	NA	NA
EIF3I	0	1	NA	NA

ERH	0	1	NA	3.0026
FBL	0	1	NA	NA
FUS	0	2	NA	0.87408
HNRNPR	0	2	NA	1.437
KIF11	0	1	NA	NA
LUC7L	0	1	NA	NA
MATR3	0	2	NA	1.0787
MRPL14	0	1	NA	NA
MRPL24	0	1	NA	NA
PARP1	0	1	NA	NA
RIOK1	0	1	NA	NA
RPL18	0	2	NA	1.0865
RPL23	0	1	NA	NA
RPL29	0	1	NA	NA
RPL4	0	2	NA	1.0278
RPL7	0	1	NA	NA
RPL8	0	2	NA	1.4455
RPL9	0	1	NA	NA
RPN1	0	1	NA	0.95
RPS25	0	2	NA	0.99559
SERBP1	0	1	NA	NA
SLC25A11	0	1	NA	NA
STAU1	0	1	NA	NA
TPM2	0	1	NA	NA
UBAP2L	0	1	NA	NA
XRCC6	0	1	NA	NA
ZC3H4	0	1	NA	NA
ZRANB2	0	1	NA	NA

Supplementary Table 3

Kinetic parameters of the mathematical model
For the ordinary differential equations see Methods section.

parameter	values (au)	biological description of the process	decadic logarithm of the sampling range (au)
k_1	9727.7	association rate of IκBα and NF-κB	[3, 5]
k_2	0.0011844	basal activation rate of IKK	[-3, -1]
k_3	0.85927	degradation rate of A20	[-2, 0]
k_4	0.22329	inactivation rate of IKK	[-1, 1]
k_5	0.0055014	degradation rate of IκBα	[-3, 0]
k_6	0.0063041	IKK-dependent degradation rate of IκBα bound to NF-κB	[-3, 0]
k_7	0.00029974	degradation rate of A20 mRNA	[-4, -2]
k_8	0.00038679	degradation rate of IκBα mRNA	[-4, -2]
k_9	0.099907	synthesis rate of A20 protein	[-2, 0]
k_{10}	12.657	TNF-dependent activation rate of IKK	[1, 3]
k_{11}	0.12952	synthesis rate of IκBα protein	[-2, 0]
k_{12}	0.098751	synthesis rate of A20 mRNA dependent on NF-κB	[-3, -1]
k_{13}	0.0015859	synthesis rate of IκBα mRNA dependent on NF-κB	[-3, -1]
k_{14}	39.284	dissociation rate of NF-κB | IκBα	[1, 3]

The parameter TNFα is either set 0 or 1 for the unstimulated or stimulated system, respectively. To account for the influence of RC3H1 overexpression on A20 mRNA and IκBα mRNA the parameters $RC3H1_{A20}$ and $RC3H1_{I\kappa B\alpha}$ are set to 2 and 1.5, respectively. To simulate the influence of siRC3H1/2 the parameters $RC3H1_{A20}$ and $RC3H1_{I\kappa B\alpha}$ are set to 0.1. Both are set to 1 otherwise. The total NF-κB concentration was estimated to be 1.4454 au. The initial concentrations are given by the unstimulated steady state.
